# Genome-wide transcriptional analysis of grapevine berry ripening reveals a set of genes similarly modulated during three seasons and the occurrence of an oxidative burst at vèraison

**DOI:** 10.1186/1471-2164-8-428

**Published:** 2007-11-22

**Authors:** Stefania Pilati, Michele Perazzolli, Andrea Malossini, Alessandro Cestaro, Lorenzo Demattè, Paolo Fontana, Antonio Dal Ri, Roberto Viola, Riccardo Velasco, Claudio Moser

**Affiliations:** 1 Department of Genetics and Molecular Biology; IASMA Research Center, Via E. Mach 1, 38010 S. Michele a/Adige (TN), Italy; 2 Department of Information and Communication Technology, University of Trento, Via Sommarive 14, 38050 Povo (TN), Italy; 3 Lorenzo Demattè: Microsoft Research-University of Trento Centre, Piazza Manci 17, 38050 Povo (TN) Italy; 4 Michele Perazzolli: SafeCrop Centre, Istituto Agrario San Michele a/Adige, Via E. Mach 1, 38010 S. Michele a/Adige (TN), Italy

## Abstract

**Background:**

Grapevine (*Vitis *species) is among the most important fruit crops in terms of cultivated area and economic impact. Despite this relevance, little is known about the transcriptional changes and the regulatory circuits underlying the biochemical and physical changes occurring during berry development.

**Results:**

Fruit ripening in the non-climacteric crop species *Vitis vinifera *L. has been investigated at the transcriptional level by the use of the Affymetrix *Vitis *GeneChip^® ^which contains approximately 14,500 unigenes. Gene expression data obtained from berries sampled before and after véraison in three growing years, were analyzed to identify genes specifically involved in fruit ripening and to investigate seasonal influences on the process. From these analyses a core set of 1477 genes was found which was similarly modulated in all seasons. We were able to separate ripening specific isoforms within gene families and to identify ripening related genes which appeared strongly regulated also by the seasonal weather conditions. Transcripts annotation by Gene Ontology vocabulary revealed five overrepresented functional categories of which cell wall organization and biogenesis, carbohydrate and secondary metabolisms and stress response were specifically induced during the ripening phase, while photosynthesis was strongly repressed. About 19% of the core gene set was characterized by genes involved in regulatory processes, such as transcription factors and transcripts related to hormonal metabolism and signal transduction. Auxin, ethylene and light emerged as the main stimuli influencing berry development. In addition, an oxidative burst, previously not detected in grapevine, characterized by rapid accumulation of H_2_O_2 _starting from véraison and by the modulation of many ROS scavenging enzymes, was observed.

**Conclusion:**

The time-course gene expression analysis of grapevine berry development has identified the occurrence of two well distinct phases along the process. The pre-véraison phase represents a reprogramming stage of the cellular metabolism, characterized by the expression of numerous genes involved in hormonal signalling and transcriptional regulation. The post-véraison phase is characterized by the onset of a ripening-specialized metabolism responsible for the phenotypic traits of the ripe berry. Between the two phases, at véraison, an oxidative burst and the concurrent modulation of the anti-oxidative enzymatic network was observed. The large number of regulatory genes we have identified represents a powerful new resource for dissecting the mechanisms of fruit ripening control in non-climacteric plants.

## Background

Grape is among the most ancient, widely cultivated fruit crops. The keen interest in the understanding of grape berry ripening is justified by the economic relevance of the quality of grapes and their processed products, such as wine, juice and dried fruit. The onset of the genomic era has brought unprecedented progress into our knowledge of *Vitis *molecular biology, and powerful genetic and genomic tools are now available or are being developed, such as mapping populations, genetic and physical maps [1,2], an extensive ESTs collection (342,576 sequences deposited at the NCBI EST database-March 19, 2007, [3]) and several tools for gene expression analysis. These resources enable us to identify grape as a model plant for studies on non-climacteric anthocyanin accumulating fruits, beside tomato, which is currently the model plant for climacteric carotenoid accumulating fruits. Grape berry development from anthesis to ripening is classically divided into three phases on the basis of chemical and morphological traits [4]: berry formation, characterized by exponential growth of the berry and accumulation of organic acids, mainly malate, in the vacuole; véraison, a transition phase during which growth declines and berries start to change colour and soften; ripening, characterized by an increase in pH, marked berry growth and accumulation of sugars, anthocyanins and flavour-enhancing compounds. Published studies based on EST analysis [5-7], differential display [8], cDNA-arrays [9] and oligo arrays [10] have shown distinct and extensive changes in the transcriptome occurring during ripening. These studies report up to 740 developmentally regulated genes mainly involved in defence, stress response, primary and secondary metabolism, berry growth and hormonal metabolism. The very recent study of Grimplet et al. [11], provides an extensive description of these functional classes investigating their tissue-specific expression in the ripe berry by means of the Affymetrix *Vitis *GeneChip^®^.

With the aim of understanding the transcriptional changes underlying grapevine berry ripening and the regulatory mechanisms acting at this level, we were able to identify 1477 genes modulated during berry development, comprising 282 regulatory factors in addition to the structural genes, by the use of the Affymetrix *Vitis *GeneChip^®^. The results, obtained from replicated experiments carried out in three growing seasons, provide also insights into the relationship between genetic and environmental effects on berry development control.

## Results and Discussion

### Grape berries phenotypic evaluations

**Figure 1:**
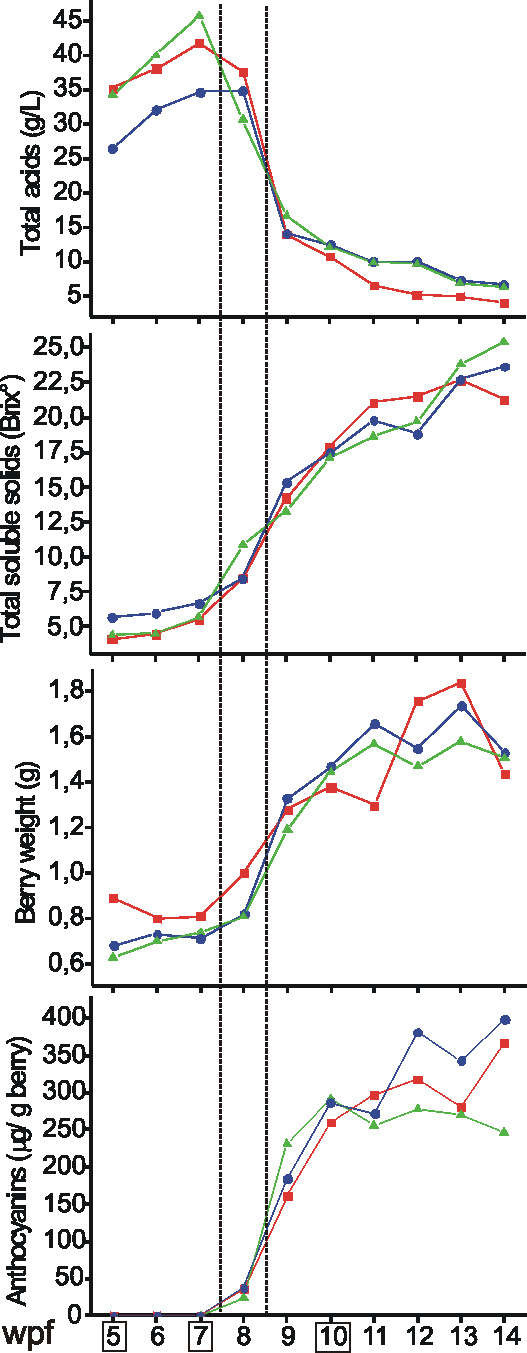
**Biochemical changes observed during Pinot Noir berry development in 2003 (red), 2005 (blue) and 2006 (green)**. Total acids (expressed as grams of tartaric acid per liter of must), total soluble solids, and anthocyanins content and berry weight averaged on a pool of 50 berries are reported. The dashed area indicates véraison. The weeks post flowering (wpf) corresponding to the samples used for the hybridization experiments are enclosed in boxes.

Figure 1 shows the changes in average berry weight, organic acids, total soluble solids and anthocyanin content of grape berries sampled weekly during the 2003, 2005 and 2006 growing seasons. The date of véraison was set at approximately 8 weeks post-flowering (wpf) in all years. In order to characterize berry ripening, we chose three distinct moments according to [12]: the last stage of berry formation, time-point A (TP A, stage E-L 33), characterized by small hard green berries still accumulating organic acids; and two stages during berry ripening, TP B (stage E-L 34), just before véraison, characterized by berries in the green state with maximum acidic content starting to soften and TP C (stage E-L 36), when the ripening process is well established and berries are growing fast, colouring and accumulating sugars. The identification of the corresponding developmental stages in the three years was based on the biochemical profiles. As average temperatures during 2003 remained constantly unusually higher from April to October [see Additional file 1], flowering and harvest occurred approximately one week earlier in 2003.

### Statistical analysis

RNA extracted in triplicate from berries harvested at three time points was used to hybridize 27 Affymetrix Vitis GeneChips^®^. Datasets from each season were analyzed separately. The quality of the biological replicates was evaluated by the R-squared coefficient [see Additional file 2], which ranged from 0.89 to 0.99. Minimum R-squared values were found for TP B samples, suggesting a higher sample heterogeneity approaching véraison, when dramatic transcriptional changes occur in less than 48 hours [10].

**Figure 2:**
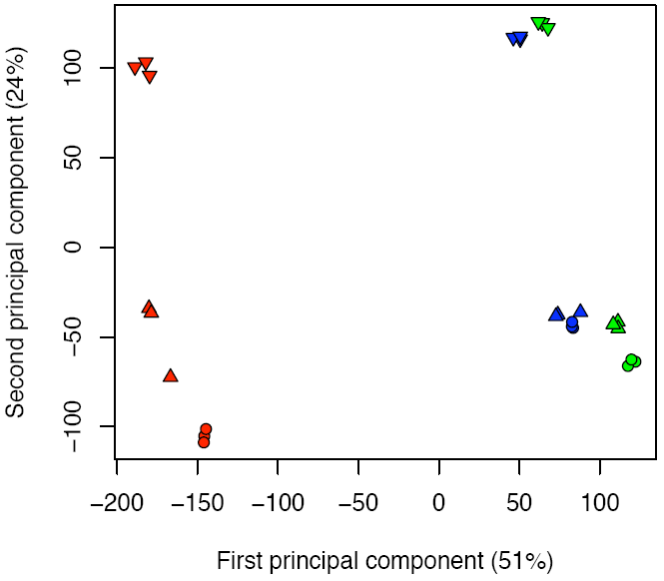
**Principal component analysis (PCA) of the 27 expression datasets**. Scaled expression data relative to 2003 (red), 2005 (blue) and 2006 (green) samples are sharply separated according to the sampling year (first principal component) and to the developmental stage (second principal component). Biological replicates relative to time point A, B and C are represented as circles, triangles and downwards triangles, respectively.

Principal Component Analysis (PCA) confirmed the uniformity of the biological replicates as the nine groups of replicates clusterised tightly (Fig. 2). The greatest variance in gene expression was found between samples from the three growing seasons, as they were separated along the first component (51% of the total variance). In particular, the largest difference was evident between the 2003 expression data compared to the 2005 and 2006, suggesting that the environmental conditions greatly affect the berry transcriptome. [see Additional file 1]. On the other hand, the second principal component (24% of the total variance) separated neatly the pre-véraison stages (TP A and TP B) from the post-véraison one (TP C), confirming previous observations of extensive transcriptional changes occurring from berry formation to berry ripening [10].

Class comparison analysis by means of Significance Analysis of Microarray [13], imposing a minimum fold change of 2 in at least one comparison (TP A vs. TP B and TP B vs. TP C), produced three sets of 2632, 4238 and 3856 differentially expressed probesets for the 2003, 2005 and 2006 samples, respectively. The size difference between the three sets likely reflects inter-seasonal biological differences. The comparison of the three datasets identified a common set of 1700 modulated transcripts and among these, 1477 transcripts with a conserved profile in the three years (Pearson's correlation coefficient >0.5 in any pairwise comparison). This 1477 core dataset, which corresponds to 9% of the chip probesets, is listed in Additional file 3. It represents a highly conserved expression network reasonably involved in an internal basic functional program, such as berry ripening.

### Annotation of the differentially expressed genes

**Figure 3:**
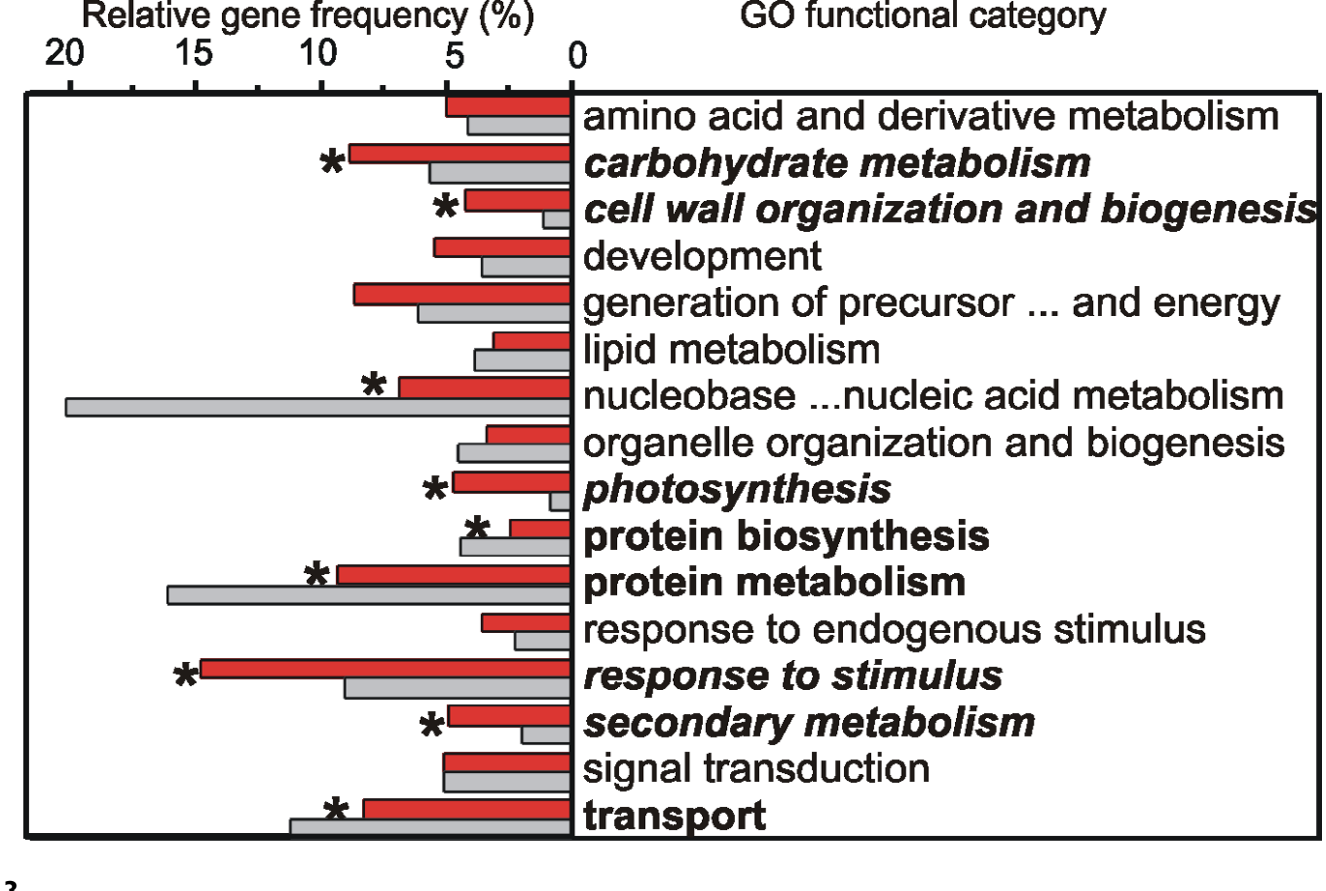
**Functional categories distribution in the core set of the modulated genes (red) and in the entire Vitis GeneChip^® ^(grey)**. Frequencies are calculated as percentage of the whole number of 'GO biological process' terms (1,825 and 21,890 in the modulated and chip sets, respectively). Nine classes, marked by an asterisk and written in bold, resulted differently represented in the modulated set compared to the entire chip after statistical analysis (p-value < 0.001). In italics are the five categories overrepresented in the modulated set.

Automatic annotation of all the Vitis GeneChip^® ^sequences was performed using the Gene Ontology (GO) classification [14]. The core set of 1477 differentially expressed genes was subsequently manually checked and integrated with additional GO 'biological process' terms. Transcripts were then grouped into 17 GO functional categories [see Additional file 3 and 4] whose distribution among the entire chip and the core set was statistically compared (Fig. 3). Nine categories were differentially represented at significant levels during berry development and five of these were overrepresented in the regulated gene core set. These were "carbohydrate metabolism", "cell wall organization and biogenesis," "response to stimulus," "secondary metabolism" and "photosynthesis," all processes that ought to be specifically regulated during grape berry development. The four underrepresented categories were "nucleobase, nucleoside, nucleotide and nucleic acid metabolism," "protein biosynthesis," "protein metabolism" and "transport." These classes may either play a minor role in the developmental program or their involvement could be restricted to a small subset of genes.

The functional class distribution of the Vitis GeneChip^® ^was also compared to that of the *Arabidopsis *genome available at TAIR ([15], [Additional file 5]). The overall agreement in the class distributions confirms the Vitis GeneChip^® ^as a reliable toolkit for the investigation of the grape expressed genome.

### Cluster analysis and functional categories modulation during berry ripening

**Figure 4:**
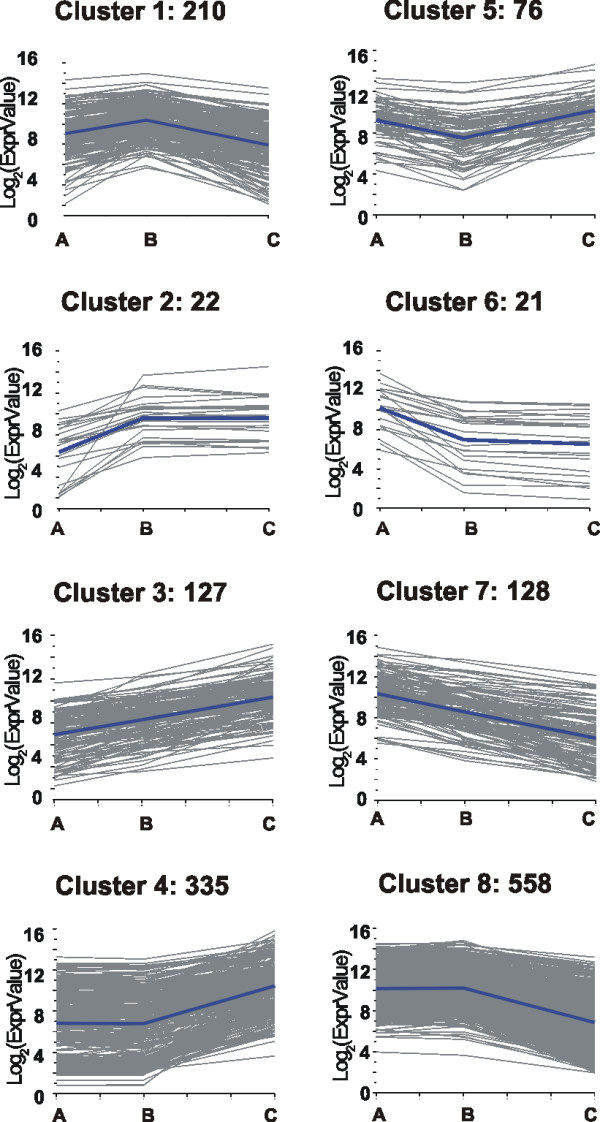
**Cluster analysis of the expression profiles of the modulated core set**. The expression profiles of the 1477 modulated genes during P. Noir berry ripening were clusterised in eight clusters which represent the minimum number of profiles considering three time points. Clusters were obtained by the k-means method using Pearson's correlation distance. The representative profile and the number of genes in each cluster are indicated.

Cluster analysis of the gene core set was based on the k-means method using Pearson's correlation distance calculated on the gene expression profiles. Transcripts were divided into eight groups representing the minimum number of profiles that could be obtained with three time-points. We observed good agreement between clustering in the three gene sets (80% of the transcripts fell in the same cluster in all seasons). We decided to use the 2005 expression data for the cluster representation (Fig. 4), due to the smaller variance among replicates [see Additional file 2]. As expected from PCA, the two most populated clusters were number 4 and 8, composed by genes modulated positively and negatively from TP B to TP C, respectively. Conversely, clusters 2 and 6, consisting of genes modulated only during the pre-véraison interval, were the less populated ones. Approximately the same number of genes was positively or negatively modulated along the whole study interval (cluster 3 and 7). Genes specifically induced around véraison should fall in cluster1 while genes with roles in early and late stages of berry development should fall in cluster 5.

**Figure 5:**
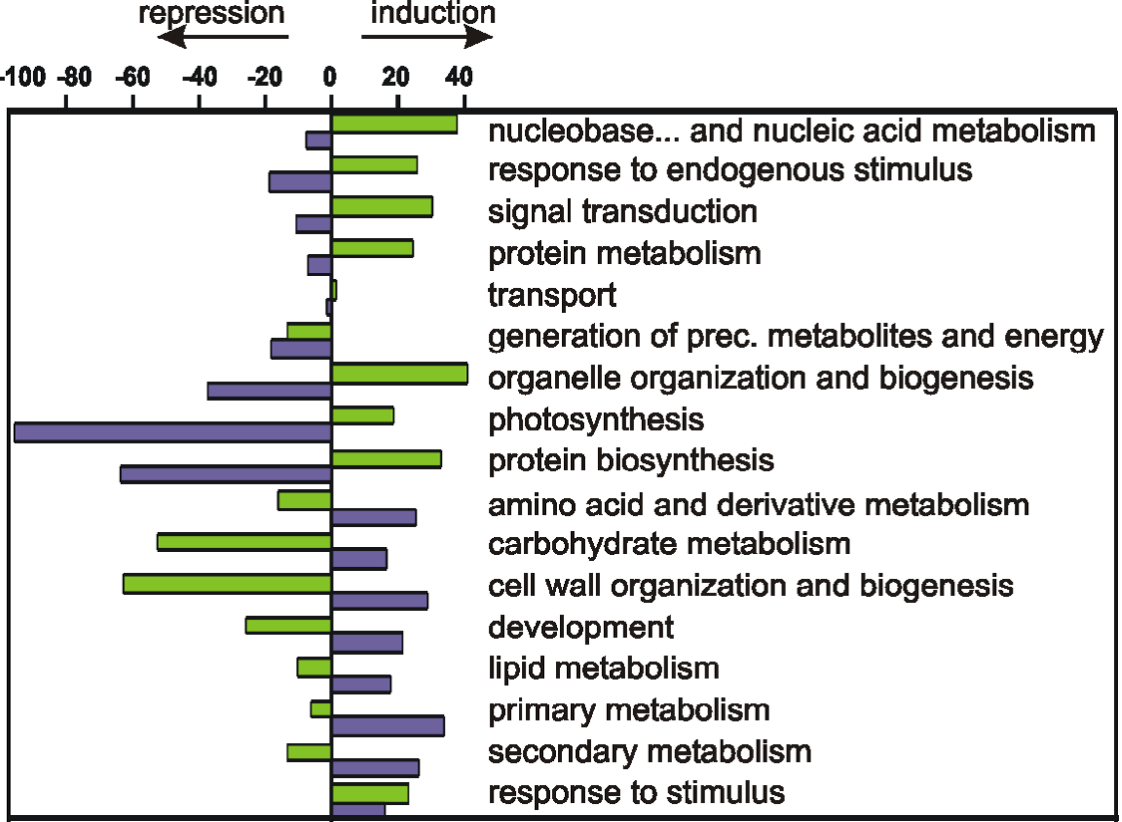
**Functional categories distribution before and after-véraison**. Induced and repressed functional categories during the first stage (from TP A to TP B, green histograms) derive from clusters 1, 2, 3 and 5, 6, 7, respectively, while categories induced and repressed during the second stage (from TP B to TP C, violet histograms) derive from clusters 3, 4, 5 and 1, 7, 8, respectively. The relative number of induced (Ni) or repressed (Nr) genes for a specific functional category was calculated with respect to the total number of induced or repressed genes in each stage. The estimate of induction or repression within each functional category was then calculated as follows: estimate of induction/repression = (Ni - Nr)/(Ni + Nr). Positive values represent an overall induction and negative values an overall repression.

Functional class distribution frequency was then calculated for each cluster and represented as histogram [Additional file 6]. To better understand the modulation of the functional classes during berry ripening, their frequencies were determined after splitting the experimental interval into two phases: one from TP A to TP B and one from TP B to TP C. The genes modulated during the first phase derived from clusters 1, 2, 3 (induced) and 5, 6, 7 (repressed), while the genes modulated during the second phase derived from clusters 3, 4, 5 (induced) and 1, 7, 8 (repressed). During the pre-véraison phase, the number of the induced genes (408) exceeded that of the repressed ones (270), while the opposite occurred during the following phase (651 induced vs. 1118 repressed). As depicted in Figure 5, all but two categories showed a general tendency towards an opposite modulation between the first and the second phase. Furthermore in the first phase a general bias towards induction for the GO categories involved in regulatory mechanisms, namely "nucleobase, nucleoside, nucleotide and nucleic acid metabolism" (mainly transcription factors), "response to endogenous stimulus" (mainly hormone metabolism), "signal transduction," and "protein metabolism" was observed. This suggests a strong cell re-programming taking place in berry cells up to véraison. During the second phase, a marked negative regulation is evident for categories involved in cell division, such as "organelle organization and biogenesis" and "protein biosynthesis," and "photosynthesis." This behaviour is coherent with the slowing down of cell replication and the loss of photosynthetic capacity. On the other hand, the classes "amino acid and derivative metabolism," "carbohydrate metabolism," "cell wall organization and biogenesis," "development," "lipid metabolism," "primary" and "secondary metabolism" display a positive regulation. This indicates the prevalence after véraison of metabolic processes involved in cell wall loosening and synthesis, sugar accumulation and synthesis and transport of metabolites responsible of grape colour and flavour. Finally, the class "response to stimulus" appeared induced in both phases, suggesting that throughout the entire berry development process the cell devotes considerable effort to face different kinds of stimuli, presumably including osmotic, oxidative and biotic stress.

### Gene regulation of grape berry ripening

Our study of the berry ripening process identified 125 transcription factors, 65 genes involved in hormone metabolism and response and 92 involved in signal transduction, corresponding to 8.5%, 4.4% and 6.2% of the regulated core set, respectively. These figures and similar data recently obtained for tomato and Citrus fruit [16,17], suggest that a consistent fraction of the modulated fruit transcriptome is devoted to the control of the developmental program. In this section we present our main contribution to the knowledge of gene regulation during berry ripening in grapevine. A restricted list of selected genes involved in the process and discussed in the text is reported in Table 1 with the specific AffyID, whereas the complete list is provided in part A of Additional file 7.

**Table 1:** Selection of genes putatively involved in the regulation of berry ripening.

**AffyID**	**TIGR TC**	**Cluster**^a^	**UniprotID**^b^	**Description**^b^	**E-value**^(b)^
**HORMONE METABOLISM AND SIGNALING**					
**AUXIN**					
*Auxin synthesis*					
1610647_at	TC40710	8	Q9FR37	Amidase (At1g08980 AtAMI1)	3,89E-152
*Auxin transport*					
1612060_at	TC48033	6	Q76DT1	AUX1-like auxin influx carrier protein	2,97E-143
1621946_at	TC50194	7	Q8H0E0	PIN1-like auxin transport protein	1,05E-51
*Aux/IAA*					
1618875_s_at	TC46557	1	Q8LAL2	IAA26 (Phytochrome-associated protein 1)	2,95E-63
1617513_at		8	Q8LAL2	IAA26 (Phytochrome-associated protein 1)	6,04E-62
1615985_at	TC42737	1	O24542	AUX22D	2,40E-69
1614660_at	TC45938	4	P13088	AUX22	6,35E-60
1621754_at	TC41728	8	Q84V38	Aux/IAA protein	9,19E-89
1615728_at	TC45614	8	Q84V38	Aux/IAA protein	0,00E+00
1611390_a_at	TC39148	8	Q8RW16	Aux/IAA protein	2,92E-98
1620512_at	TC46080	8	Q9ZSY8	IAA27 (Phytochrome-associated protein 2)	9,42E-86
1613468_at	TC38800	8	Q8LSK7	Auxin-regulated protein (IAA1)	3,38E-99
*SAUR*					
1621201_at	TC41864	1	Q6ZKQ7	Auxin-induced protein (SAUR)	5,99E-15
*GH3*					
1610880_s_at	CF371851	8	O22190	Indole-3-acetic acid-amido synthetase GH3.3	1,78E-33
1612001_s_at	TC43261	8	Q6QUQ3	Auxin and ethylene responsive GH3-like protein	6,67E-111
1607503_s_at	TC38370	8	Q52QX4	Auxin-repressed protein-like protein ARP1	2,50E-48
*Other auxin responsive proteins*					
1619856_at	TC45354	1	O48629	Putative auxin-repressed protein (dormancy)	6,41E-44
1621185_s_at	TC45354	1	O48629	Putative auxin-repressed protein (dormancy)	6,41E-44
1613204_at	TC38341	2	O49235	2.4-D inducible glutathione S-transferase	1,74E-98
1619913_at	TC46727	4	Q9SV71	auxin-responsive protein	5,97E-116
*ARF (transcription factor)*					
1612180_at	TC51304	8	Q6L8T9	Auxin response factor 5	1,24E-64
1616225_at	TC44210	8	Q9C5W9	Auxin response factor 18	3,69E-88
*Auxin receptor*					
1612090_s_at	TC45186	8	Q9ZRA4	Auxin-binding protein ABP19a precursor	1,75E-88
**ETHYLENE**					
*Ethylene synthesis*					
1615952_s_at	TC38453	1	Q84X67	1-aminocyclopropane-1-carboxylic acid oxidase 1. ACO	1,20E-167
1622308_at	TC40451	8	Q8S933	1-aminocyclopropane-1-carboxylate synthase. ACC synthase	3,92E-68
1612699_at	BQ798614	8	Q9XIA5	Similar to ethylene-forming-enzyme-like dioxygenase	5,03E-29
*Ethylene responsive transcription factors*					
1617012_at	TC45046	3	Q8S9H4	Tomato Ethylene response factor 1	5,10E-61
1611910_s_at	-	8	Q6TKQ3	Putative ethylene response factor ERF3b	7,26E-124
1609990_at	TC39828	8	Q6TKQ3	Putative ethylene response factor ERF3b	1,45E-123
1620278_at	TC46710	3	Q6J9Q4	Putative AP2/EREBP transcription factor	2,74E-64
1621270_at	TC47575	8	Q9LYD3	Putative AP2/EREBP transcription factor (DREB3)	3,34E-63
*Ethylene induced genes*					
1618213_at	-	1	Q9SWV2	ER6 protein (Fragment)	3,89E-18
1619086_at	TC39832	8	Q93W91	Putative ER6 protein	2,55E-51
1609780_at	TC47621	8	Q94E74	Putative ER6 protein. Ethylene-responsive protein	5,74E-33
1613123_at	CF215236	1	Q8S3D1	bHLH transcription factor (tomato ER33 homolog)	1,04E-61
1613177_at	TC47351	8	Q9LSP8	Ethylene-induced calmodulin-binding transcription activator	3,76E-94
1622850_at	TC49495	3	O48631	Ethylene-forming-enzyme-like dioxygenase (tomato E8 homolog)	7,27E-78
*Pathogenesis related genes putatively induced by ethylene*					
1611058_at	TC47119	4	Q7XAJ6	Putative pathogenesis related protein 1 precursor	1,55E-73
1618835_s_at	TC38693	4	O81228	PR-4 type protein	1,62E-80
1622360_at	TC40938	4	Q42966	NtNitrilase 4B (EC 3.5.5.1)	1,05E-135
**ABSCISIC ACID**					
*Abscisic acid synthesis*					
1607029_at	TC42536	4	Q8LP14	Nine-cis-epoxycarotenoid dioxygenase4	1,32E-51
*Abscisic acid responsive proteins*					
1618211_at	TC47430	4	P93615	ABA induced plasma membrane protein PM 19	1,37E-60
1614372_at	TC47836	4	Q6H5X2	ABA-responsive protein-like	3,03E-61
1614788_at	TC45479	4	Q4VT48	Dehydrin	2,92E-69
1621592_s_at	TC45479	4	Q4VT48	Dehydrin	2,92E-69
1606669_s_at	-	4	Q5PXH0	Aquaporin (PIP2_1)	9,87E-74
1615808_s_at	TC38121	4	Q5PXH0	Aquaporin (PIP2_1)	2,63E-162
1610982_at	TC38138	8	O24049	Major intrinsic protein C, aquaporin	6,31E-141
1614916_at	TC38138	8	O24049	Major intrinsic protein C, aquaporin	6,31E-141
1612244_s_at	TC38281	8	Q9M7B0	Putative aquaporin PIP2-2	2,40E-158
**BRASSINOSTEROIDS**					
*Brassinosteroid synthesis*					
1608099_at	TC40368	8	Q43147	Cyt. P450 85A1, C6-oxidase, Dwarf protein	2,89E-74
*Brassinosteroid receptor*					
1612516_at	TC47080	1	Q76FZ8	Brassinosteroid receptor	0,00E+00
**GIBBERELLINS**					
1610607_at	TC40495	5	Q6NMQ7	Gibberellin-responsive protein (At1g74670)	1,04E-28
1609893_at	TC38517	8	O24040	LtCOR11 (Gibberellin regulated protein)	1,62E-36
1618503_at	TC47765	8	Q75V70	Gibberellin 2-oxidase 1	1,50E-69
**CYTOKININS**					
1612955_at	TC47637	4	Q71BZ3	Type-A response regulator	3,73E-11
1619945_at	CB345883	8	Q39636	CR9 protein. cytokinin-repressed gene	3,12E-30
1620306_at	TC47826	8	Q67YU0	Cytokinin oxidase 5 (cytokinin degradation)	6,29E-113
**LIGHT STIMULUS**					
*Circadian rhythm*					
1608397_at	TC43962	8	Q6LA43	Two-component response regulator-like APRR2 (TOC2 protein)	2,77E-34
1617059_at	TC42407	4	Q9CAV6	WNK1 kinase	8,07E-26
1609874_at	TC43754	8	Q6R3R2	CONSTANS-like protein CO1	1,80E-76
1614417_at	TC47511	3	O81834	Transcription factor Constans-like family (At4g27310)	1,32E-32
1613978_at	TC49120	3	Q5GA60	Putative EARLY flowering 4 protein	1,22E-30
*Flowering control*					
1614189_at	TC47473	8	Q76CC3	Flowering locus T	1,68E-66
*Transcription factors*					
1620877_at	TC40824	8	Q948G4	Putative GATA-1 zinc finger protein	1,14E-31
*Hormone and light signals*					
1612955_at	TC47637	4	Q71BZ3	Type-A response regulator	3,73E-11
1616694_at	TC46350	7	Q94KS0	Histidine-containing phosphotransfer protein	1,96E-57
1614802_at	TC41815	7	Q94KS0	Histidine-containing phosphotransfer protein	6,27E-59
1617513_at		8	Q8LAL2	IAA26 (Phytochrome-associated protein 1)	6,04E-62
1618875_s_at	TC46557	1	Q8LAL2	IAA26 (Phytochrome-associated protein 1)	2,95E-63
1620512_at	TC46080	8	Q9ZSY8	IAA27 (Phytochrome-associated protein 2)	9,42E-86
*Other light regulated genes*					
1614237_a_at	TC38178	1	Q7XAB8	Light-regulated chloroplast-localized protein	5,16E-23
1618107_at	TC46244	1	Q93WJ7	Leaf Lethal Spot 1-like protein	0,00E+00
1614720_at	TC46244	1	Q93WJ7	Leaf Lethal Spot 1-like protein	0,00E+00
1616605_at	TC45785	1	Q8W5A3	Lethal leaf spot 1-like protein	0,00E+00
1617173_s_at		4	Q6T6I9	ELIP Early light-induced protein	3,88E-54
1610360_at	TC38699	8	Q6Q9W1	Ultraviolet-B-repressible protein	2,47E-36
1620292_at	TC38866	8	Q6Q9W1	Ultraviolet-B-repressible protein	2,00E-33
**TRANSCRIPTION FACTORS**					
*MADS-BOX TF*					
1621827_at	TC39767	1	Q6UGQ6	MADS-box protein 15	1,95E-47
1621836_at	TC45777	7	Q8LLQ9	MADS-box protein 5	6,25E-119
1607973_at	TC38620	8	Q9ZTV9	MADS1-like	4,87E-107
1613748_at	TC40277	8	Q8LLR2	*Vitis*. MADS-box protein 2, Ap1-like, MADS-RIN-like	1,73E-77
*MYB TF*					
1611920_at	TC40303	1	Q9M9A3	F27J15.20 (Hypothetical protein) (MYB transcription factor)	3,83E-84
1618260_s_at	TC42111	3	Q8L5N7	Myb-related transcription factor VlMYBB1-2, similar to	4,47E-43
1620959_s_at	TC48485	4	Q6L973	Myb-related transcription factor VvMYBA1	2,04E-119
1609612_at	TC47332	7	Q688D6	Putative myb transcription factor (MYB16 protein)	3,17E-21
1612686_at	TC48806	8	Q5NDD2	Putative MYB transcription factor	6,03E-84
1613486_at	TC45686	8	O04544	F20P5.26 protein (At1g70000) (MYB transcription factor)	1,60E-74
1614932_at	TC48806	8	Q5NDD2	Putative MYB transcription factor	6,03E-84
1618514_at	TC51437	8	O22059	CPC (Putative MYB family transcription factor)	4,70E-11
1619201_at	TC49224	8	Q8GV05	TRIPTYCHON (MYB transcription factor)	2,93E-29
*NAC TF*					
1621448_at	TC47141	2	Q52QR1	NAC domain protein NAC5	1,39E-27
1609172_at	TC39120	3	Q52QR2	NAC domain protein NAC4	1,10E-126
1606678_at	TC42489	4	Q8LKN7	Nam-like protein 17 (Fragment)	6,53E-19
1607120_at	TC46243	4	Q84K00	NAC-domain containing protein 78 (ANAC078)	1,43E-34
1621255_at	TC41700	4	Q8LRL4	Nam-like protein 11	1,85E-46
*WRKY TF*					
1609130_at	TC46341	3	Q9FGZ4	WRKY DNA-binding protein 48	1,62E-56
1622333_at	TC40428	3	O22900	WRKY DNA-binding protein 23	5,68E-46
1611285_s_at	CA809190	7	Q6IEL3	WRKY transcription factor 68	1,84E-39
*Homeotic TF*					
1616863_at	TC41982	1	Q8LLD9	BEL1-related homeotic protein 29 (Fragment)	1,20E-78
1618774_at	TC40997	1	Q4VPE9	Lateral organ boundaries-like 1 (Fragment).	6,51E-52
1611583_at	TC40246	3	Q9FJ90	Similarity to AP2 domain transcription factor	2,85E-55
1609295_at	TC47034	3	P46897	Homeobox-leucine zipper protein ATHB-7	2,41E-53
1620170_at	TC42225	7	Q9XHC9	APETALA2 protein homolog HAP2	4,04E-46
1607122_at	TC51295	8	Q546G6	Homeodomain-leucine zipper protein HAT22	4,82E-15
1607284_at	TC40589	8	Q7Y0Z9	Bell-like homeodomain protein 3 (Fragment)	3,12E-114
1617931_at	TC40185	8	Q9M276	Homeobox-leucine zipper protein ATHB-12	4,41E-34
1615625_at	TC39581	8	O04136	Homeobox protein knotted-1 like 3 (KNAP3)	6,59E-86
1613036_at	TC39310	4	Q56R05	Putative pollen specific LIM domain-containing protein	2,68E-90
1606561_at	TC41613	7	O81384	Putative basic helix-loop-helix DNA binding protein TCP2	1,25E-20
1611070_at	TC49539	7	Q8L8A5	GRF1-interacting factor 1 (Hypothetical protein At5g28640)	7,97E-43
1606591_at	TC41789	8	Q8RU28	Putative SHORT-ROOT (SHR) protein (Fragment)	1,28E-38
1610187_a_at	TC47632	8	Q9FL03	SCARECROW gene regulator	1,11E-110
1619334_at	TC47632	8	Q9FL03	SCARECROW gene regulator	1,11E-110
1612362_at	TC41970	8	Q6SS00	YABBY-like transcription factor GRAMINIFOLIA	3,06E-69
1613445_at	TC40338	8	Q6XX20	Mutant cincinnata	3,64E-16
1614851_s_at	TC40029	8	Q9SIV3	Expressed protein (GPRI1) (Golden2-like protein 1)	2,10E-39
1617877_at	TC41303	8	Q8SBC9	Transcription factor LIM	1,71E-92

^(a)^: cluster number as reported in Figure 4.

^(b)^: Uniprot ID, description and E-value of the annotation results obtained by 'blast' and 'GORetriever' analysis.

#### Hormones metabolism and signalling

Amongst the genes related to hormone metabolism in the core set, those related to auxin and ethylene were the most represented, followed by those related to abscisic acid and brassinosteroids. Few genes related to cytokinines, gibberellins or jasmonic acid were found. These observations are consistent with our knowledge of the relative importance of these signalling pathways during grape berry development [18,19].

In fruits from both climacteric and non-climacteric species, auxins are known to be more abundant immediately after ovary fertilization and to mediate important signals for the onset of fruit development [20,21]. In grape, it has been generally accepted that indole-3-acetic acid (IAA) content reaches its maximal level just after anthesis and then declines to very low levels in the ripe fruit [22], even if a recent study on *Vitis vinifera *cv Cabernet Sauvignon did not confirm it [23]. The expression of genes involved in cell wall metabolism (GRIP4), anthocyanin synthesis (CHS and UFGT) and sugar storage (GIN1) and the accumulation of the correlated metabolites were all shown to be retarded by auxin treatments at véraison [19].

In the present study, we have identified a gene homologous to the *Arabidopsis *amidase AtAMI1, which *in vitro *synthesizes IAA from indole-3-acetamide [24], and its descending profile during ripening agrees well with the decline in IAA levels observed in *Vitis labrusca *berries [22]. We have also found a gene homologous to an *Arabidopsis *IAA-amino acid synthetase which may contribute to IAA intracellular homeostasis via amino acid conjugation of excess IAA [25]. Two auxin carriers (an AUX1-like and a PIN1-like), putatively mediating auxin efflux, were also expressed before véraison.

We detected numerous auxin-regulated genes belonging to the Aux/IAA, Small Auxin-Up RNA and GH3 gene families. These families are characterized by the presence of an Auxin Responsive Element in the promoter region of their members, recognized by specific Auxin Response Factors (ARFs). Two ARFs, ARF5 and ARF18 and an auxin receptor of the ABP family were found expressed during the pre-véraison stage and then steeply repressed during ripening. With the exception of two Aux/IAA proteins, which were induced during ripening, all the other transcripts presented profiles with either a peak of expression around véraison or a decreasing profile after it. The auxin responsive genes seem therefore timely co-ordinated with the cellular auxin level, reflecting its prevalent role in the first stages of berry development. We have also detected a gene (1612001_s_at) homologous to a *Capsicum chinense *GH3 gene, which has been proposed as point of convergence between auxin and ethylene signals in non-climacteric fruit development [26]. While in pepper this GH3 transcript is up-regulated during ripening due to its ethylene-inducibility, our analyses showed a steep repression of the grape homologue after TP B.

The role of ethylene during development and ripening of some non-climacteric fruits was suggested since the 1970s and has recently been well documented [27-29]. In grape, a small and transient increase of endogenous ethylene production occurs just before véraison, in concomitance with an increase in 1-aminocyclopropane-1-carboxylic acid (ACC) oxidase activity [29]. Our results well suit these reports, as we observed a peak of expression of ACC oxidase around véraison, while ACC synthase and a putative ethylene-forming-enzyme dioxygenase, detected with similar profile also by others [10], were expressed until véraison and then repressed.

We were not able to identify any receptor or kinase involved in ethylene signal transduction in grape, within the modulated gene set. Instead, we detected four transcription factors of the Ethylene Responsive Factors (ERFs) and AP2/EREBP families. The transcript homologous to tomato LeERF1 and a putative AP2/EREBP transcription factor were induced during berry ripening, while the homologous of ERF3b and another putative AP2/EREBP transcription factor were repressed. Considering that in tomato, tobacco and *Arabidopsis*, ERF1 genes are induced by ethylene and wounding and positively regulate downstream genes involved in biotic and abiotic stresses [30], the induced transcription factors that we have identified could be involved in ethylene mediated responses to plant stresses in grape as well [31,32]. The *Vitis *homologues to osmotin, PR-1 and PR-4, downstream genes known to be regulated by ERFs in the species above mentioned, were indeed found to be induced in our study, suggesting that they could be under the same regulatory mechanism. The homologous of tobacco nitrilase NtNIT4B [33], another gene related to the transcriptional control of ethylene responsive defence genes, was found to be induced during ripening in our analysis as well. We could not thus exclude that an additional role of ethylene during grape berry development is related to biotic stress response.

Sequence annotation revealed the modulation of some transcripts homologous to the tomato genes ER6 and ER33, whose functions are still unknown but are reported to be induced by ethylene during tomato ripening [34]. In our experiment, however, these genes did not show strong modulation around véraison and they were all repressed during ripening. On the other hand, a transcript homologous to tomato E8, was found induced in grape, in agreement with tomato and strawberry gene expression data [35,36]. Although E8 resembles ACC synthase structure, it apparently exerts a negative control on ethylene biosynthesis [35] and its expression seems affected by the homeotic transcription factor LeMADS-RIN [37]. E8 similar modulation in both climacteric and non-climacteric fruits raises the possibility of a common regulatory mechanism for ethylene homeostasis control in fleshy fruit ripening.

The phytohormone abscisic acid (ABA) regulates processes such as floral transition, embryo maturation, seed development and tolerance to abiotic and biotic stress [38]. In grape berries, ABA begins to accumulate at véraison and is thus considered a good candidate for triggering berry ripening [18]. The present analysis found three ABA-related transcripts induced after véraison: one is homologous to the pea nine-cis-epoxycarotenoid dioxygenase 4 and thus putatively involved in ABA synthesis; the other two are homologous to two ABA responsive proteins. Recently, a *Vitis *dehydrin, DHN1 [39], and some *Arabidopsis *plasma membrane aquaporins (PIPs) [40], have been reported to be regulated by ABA and involved in stress and senescence, respectively. Interestingly, in our analysis we found VvDHN1 and numerous VvPIP isoforms modulated in a ripening specific way (clusters 4 and 8).

Brassinosteroids accumulate during fruit development and seem to play a key role in determining the onset of ripening in fleshy fruits [23,41]. Three *Vitis *genes involved in brassinosteroid biosynthesis and sensing have been recently cloned and transcriptionally characterized during berry development [23]. Our analysis identified VvBR6OX1, which converts 6-deoxocastasterone to castasterone, the only bioactive brassinosteroid detected in grape [23], and VvBRI1, a brassinosteroid receptor. Their expression profiles were characterized by a peak of induction at TP B, in agreement with previous data [23].

#### Light stimulus

We identified numerous transcripts putatively involved in the circadian rhythm oscillatory system. In particular, one member of the His-to-Asp two-component signal transduction family, homologous to *Arabidopsis *APRR2, and one transcription factor of the Constans-like family, showed a decreasing profile after véraison, while a kinase homologous to WNK1 [42], and two transcription factors, belonging to the Constans-like and Early flowering family respectively [43], displayed a positive regulation, more marked after véraison. A transcript homologous to Flowering locus T, putatively coordinating the circadian rhythm with the flowering switch [44], appeared expressed until TP B, thus suggesting its involvement in signalling circuits during early berry development.

Some transcripts putatively involved in light and hormonal signalling cross-talk were also identified. IAA26/phytochrome associated protein 1 and IAA27/phytochrome associated protein 2, involved in light and auxin signal transduction, were down regulated after véraison. Members of the His-to-Asp family, such as Type A response regulators, pseudo response regulators and histidine-containing phosphotransfer proteins, are known to mediate the responses to light, osmotic, cytokinin and ethylene stimuli (reviewed in [45]) and were found modulated during the process of berry development.

#### Transcription factors

In our study, we identified 125 transcription factors, among which members of the myb, MADS-box, NAC and WRKY families and some homeotic and development-specific genes.

Within the myb family, we found two previously isolated *Vitis *genes: VvmybA1, which showed a ripening-specific profile highly correlated to those of UDP-glucose:flavonoid 3-O-glucosyltransferase, glutathione-S-transferase 4 and the caffeoyl-CoA O-methyltransferase as described in [46], and a VlmybB-like gene, which was induced during berry development as reported for *Vitis labruscana *[47]. The recently characterized grape Vvmyb5A, proposed to be involved in the regulation of the phenylpropanoid pathway [48], was found to be expressed invariantly at low levels in all years during this study interval (1621471_s_at), thus not supporting a ripening-specific function for this gene.

Concerning the MADS-box family of transcription factors, only one previously characterized grape gene was present in the regulated set, namely VvMADS5, which was highly induced during early berry development, in agreement with [49]. Both VvMADS1 (1619742_at) and VvMADS4 (1614965_at), which were previously reported as abundant transcription factors involved in berry development [49,50], were highly expressed at all time-points in our experiment and thus not included in the regulated set.

1613748_at could represent the homologue of tomato LeMADS-rin (E-value = 8.2e-50, 68% identity), which is part of the developmental signalling system that initiates ripening in this fruit [51]. However, while LeMADS-rin is induced from breaker stage to ripeness, its putative grape homologue was slightly induced until véraison and then showed a decreasing profile. This behaviour could either suggest that the grape transcript is not the homologue of LeMADS-rin or that its role is different in non-climacteric fruits.

Five transcripts with homology to NAC transcription factors appeared modulated, all in a positive way in the study interval. This is of interest as recent transcriptome studies report the regulation of NAC transcription factors family not only during biotic and abiotic stress responses, but also in many other processes, such as fruit development and ABA signalling (reviewed in [52]).

Three transcripts homologous to the *Arabidopsis *WRKY family were also modulated. In plants, the WRKY family is quite large (74 members in *Arabidopsis *and 90 in rice) and its members participate in numerous cellular processes, such as defence and hormonal signalling, developmental programs and fruit maturation (reviewed in [53]).

Nine putative homeotic genes previously identified in floral development studies and 11 transcripts involved in other morphological and/or developmental processes were modulated in our study although their connection to fruit development has not yet been reported and will need further investigation. 1606591_at and 1619334_at correspond to two transcriptional regulators, SHORT ROOT and SCARECROW, which in *Arabidopsis *are both implicated in developmental root layers specification [54]. As they were co-expressed in our analysis, they might interact in berry as well.

Though not present in the regulated gene set, 1616455_s_at, corresponding to the grape VvMSA transcription factor ought to be mentioned. VvMSA is induced by sugar and ABA and positively regulates VvHT1 expression [55,56]. In our study, 1616455_s_at was very highly expressed at all three time-points, in agreement with previous proteomic and gene expression studies [7,55,57].

### Genes responsible for berry phenotypic traits and stress response

Berry development involves major transcriptional changes of genes directly responsible for the biochemical and phenotypical characteristics of each growth stage. Several protein families linked to these phenotypic traits have been expansively described in past studies focussed on single or small gene sets [58] and recently in a genome-wide study [11]. The present work provides an extensive description of gene expression during ripening, reporting for the first time the occurrence of an oxidative burst immediately after véraison. The complete list of the genes discussed in the text is in part B of Additional file 7, ordered by GO functional category; for clarity, they are further divided according to more specific GO metabolisms, when possible.

#### Cell wall metabolism

The previously described involvement of specific gene families in berry growth and softening [59] was confirmed in the present study. Among these, there were pectin modifying enzymes (methylesterase, invertase/pectin methylesterase inhibitor, pectate lyase, pectinesterase), extensins and expansins, grape ripening-induced proteins (GRIP 3, 13, 15, 22 and 28) and xyloglucan endotransglycosylases (XETs) [8,59]. In particular, 10 members of the XET family were modulated and four of these very strongly during ripening (1613415_at, 1615809_at, 1619355_at, 1621251_s_at, cluster 4). These induced isoforms are homologous to the tomato SIXTH5, 6 and 8 XET isoforms, recently defined as Group 3 XETs [60]. Although from the analysis of the tomato fruit cDNA libraries only SIXTH5 was found expressed [60], our array-based gene expression analysis suggests that all the three isoforms of group 3 could be involved in fruit development sharing a common function. *Vitis *XET isoforms have been proposed to be implied in brassinosteroids mediated berry cell wall softening [23].

#### Stress response

The present analysis identified 270 transcripts related to plant stress perception and response which fall into the "response to stimulus" GO category.

Sixty-nine transcripts were involved in plant biotic stress response, and they were mostly accumulating during ripening. The induction of defence genes including GRIPs (GRIP 22, 28, 31, 32, 51, 61), thaumatins, lipid transfer proteins, chitinases, γ-thionins and pathogenesis related proteins during berry ripening, confirmed previous results [8].

A particular kind of stress response which has been reported during fruit development in several species such as tomato [61], strawberry [20], avocado [62], pineapple [63] and pear [64], is the response to oxidative stress. In recent years a dual role for reactive oxygen species (ROS) in plants has been identified: toxic by-products of the aerobic metabolism and important regulators of growth, development and defence reactions. A complex network of genes finely tuning ROS concentration as the result of their production and scavenging has been documented [65].

**Figure 6:**
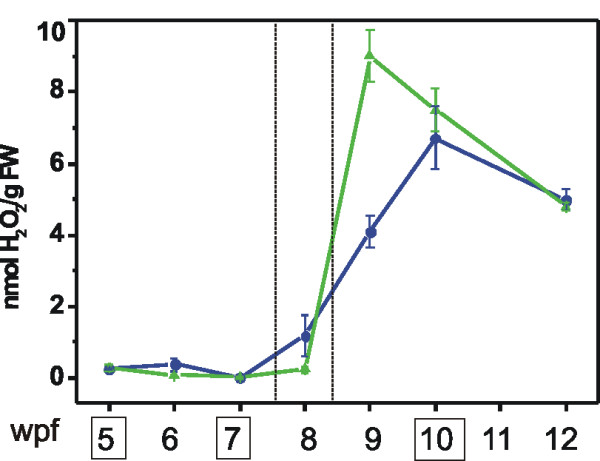
**H_2_O_2 _content during berry ripening in 2005 (blue) and 2006 (green) samples**. Data are means ± SE of three replicates. The weeks post flowering (wpf) corresponding to the samples used for the hybridization experiments are enclosed in boxes. Véraison time is delimited within dotted lines.

**Figure 7:**
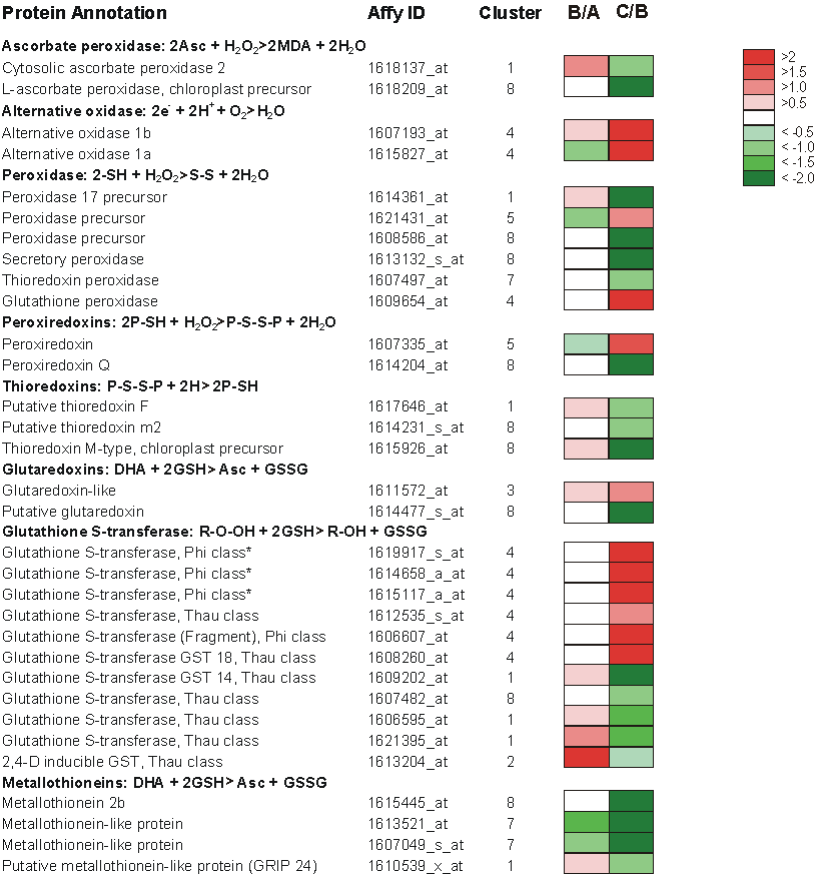
**Genes involved in the response to oxidative stress modulated during ripening**. List of the ROS-scavenging genes found in the modulated core set and comparative analysis of their expression in the three sampling TPs. Log_2 _ratios of the expression values observed in TPs A, B and C (B/A and C/B) are visualized by a colour scale, where red indicates an increase and green a decrease. For each functional group the corresponding enzymatic reaction is reported. * refer to probesets probably corresponding to the same transcript.

The occurrence of oxidative stress during grape berry development has been rather controversial: catalase was identified among the abundant proteins in the ripe berry [57], but at the transcriptional level the typical oxidative stress markers seemed absent or negatively regulated [10]. The present study clearly indicated that oxidative processes take place also during berry ripening and that an enzyme-mediated scavenging system is activated. Berry H_2_O_2 _content increased significantly in correspondence of the véraison stage, reaching its maximum one-two weeks after, and then decreasing at a slower pace towards harvest time (Fig. 6). At least 32 transcripts of the modulated core set appeared involved in the enzymatic detoxification from H_2_O_2 _and the other ROS species which accumulate in the cell starting from véraison (Fig. 7). Some of them, such as ascorbate peroxidase, glutathione peroxidase and peroxiredoxins, catalyze the direct reduction of hydrogen peroxide to water; the others (thioredoxins, glutaredoxins, glutathione-S-transferases and metallothioneins) regulate the balance of the oxidized and reduced forms of the antioxidants ascorbic acid and glutathione. Although the regulation of the detoxifying enzymes must be very complex, isozyme specific, and occurring at different levels (transcriptional- and post-transcriptional regulation, subcellular compartimentalization, etc) as previously shown for some of them [61], most of the enzymes shown in Figure 7 appeared to be unchanged from TP A to TP B and be strongly modulated from TP B to TP C. Further studies are needed to address how ROS molecules are taking part to these regulatory circuits and what is the main reason of ROS accumulation at véraison. The superoxide dismutase (SOD) and catalase (CAT) transcripts which also codify for two important detoxifying enzymes were not significantly modulated in our study. The CAT gene (1618705_s_at) was highly expressed in all the three TPs, the SOD encoding transcripts (5 probes) showed very different expression levels but no one fell into the modulated core set.

The Nudix gene family may also be involved in the oxidative stress response, as three transcripts were found modulated (one in cluster 3 and two in cluster 8). In *Arabidopsis*, Nudix hydrolases appear to be involved in the sanitization of the oxidized nucleotide pool accumulating during oxidative stress [66].

Ten genes involved in programmed cell death (PCD) appeared to be modulated. In particular, three transcripts with putative function of PCD suppression are grouped in cluster 1, while a putative death associated protein is induced after véraison (cluster 4). In addition, listed in the protein metabolism category there are two cysteine proteases (cluster 4) and a cystatin-like gene (cluster 5). Our data are insufficient to draw any conclusion, however they may suggest the occurrence of a PCD process in grape berry during ripening, as highlighted for strawberry [20].

#### Sugar/acid ratio and water balance

Our results support the model of sugar phloem unloading via an apoplastic pathway by means of plasma membrane sucrose and hexoses transporters as proposed by [67] and recently confirmed by [68,69]. Four sugar transporters were found modulated: the hexose transporter VvHT6 (1615257_at and 1619691_at) was positively modulated during ripening (cluster 3), as previously described [10]; interestingly, a transcript homologous to a sugar transporter isolated in Citrus fruit was also induced while the forth transcript, which is homologous to a sugar transporter from tomato root is repressed. The sucrose transporter VvSUC27 appeared dramatically down-regulated after véraison, as already reported in the cv. Syrah [70], while, VvSUC11 and VvSUC12, transporters with a ripening specific expression profile [70], were highly expressed at all time points in our experiment.

There is evidence that during berry ripening, sucrose is hydrolysed in the cell wall and then re-synthesised in the cytoplasm following hexose uptake [69]. Our data corroborate this mechanism as three sucrose-phosphate synthases (cluster 3 and 4), a sucrose synthase (cluster 4), a sucrose-6-phosphate phosphatase (cluster 4), a UDP-sugar pyrophosphorylase (cluster 2) and a beta-phosphoglucomutase (cluster 4) were all up-regulated during ripening. Vacuolar invertases, VvGIN1 and VvGIN2, both resulted highly expressed until véraison (cluster 8), as previously described [57,71]. The existence of 'futile cycles' of sucrose synthesis and breakdown has been demonstrated in other fruits, such as tomato, where the relative rate of synthesis and breakdown cycles controls the rate of sugar and starch accumulation [72].

The early expression of a phosphoenolpyruvate carboxylase isoforms (cluster 8) well correlated with the rate of malate accumulation during the first phase of berry development. Furthermore, a vacuolar pyrophosphatase (1614834_at) putatively involved in malate vacuolar uptake was expressed early and then induced during ripening (cluster 5), as previously observed [73]. At véraison, when malate decompartimentalization and breakdown take place [67], induction of malate degrading enzymes, such as malic enzyme and phosphoenolpyruvate carboxykinase (cluster 1) and mitochondrial malate dehydrogenase (cluster 8) was observed. The profiles observed for grape alcohol dehydrogenase 2 and a putative short chain alcohol dehydrogenase (cluster 4 and 5, respectively) and three isoforms of aldehyde dehydrogenase (one in cluster 4 and two in cluster 8) may be indicative of a shift to an aerobic fermentative metabolism during ripening [74]. The alpha subunit of the pyruvate dehydrogenase complex was induced after véraison (cluster 5); however, this enzyme is known to be highly regulated post-translationally, allosterically by NADH and covalently by reversible phosphorylation. Our analysis identified two mitochondrial pyruvate dehydrogenase kinase isoforms, one of which induced in a ripening-specific way. Two enzymes of the glycolysis/gluconeogenesis pathways, such as enolase and glyceraldehyde-3-phosphate dehydrogenase (cluster 5 and 4, respectively) were induced during ripening, suggesting their involvement into the acid/sugar interconversion and/or the fermentative metabolism.

About 90% of ripe berries fresh weight is comprised of water whose transport into the berry vacuole is mediated by aquaporins. In our study we detected 13 transcripts coding for aquaporin isoforms: eight belong to the plasma membrane intrinsic protein family, four to the tonoplast intrinsic protein family and one to the NOD26-like intrinsic protein family. All isoforms were highly expressed in the pre-véraison phase and three of them, coding for the putative aquaporin PIP2-1, were also induced during ripening. A similar modulation has been recently observed in Citrus, although the induced isoforms during fruit development, belong to the TIP family [16].

#### Secondary metabolism

The "secondary metabolism" GO functional category included genes involved in the synthesis of aromatic and volatile compounds, such as terpenes and benzenoid compounds; antioxidant compounds, such as polyphenols and vitamin E, and genes of the phenylpropanoid pathway. Due to its importance for berry quality traits, we restricted our analysis to the latter pathway. Most structural genes of this branched pathway, which starts with phenylalanine ammonia lyase, were present among the regulated genes of the current study. During ripening, the genes coding for the enzymes acting in flavonols synthesis (flavonol synthase, cluster 3), stilbenes synthesis (three isoforms of stilbene synthase, cluster 4) and anthocyanins synthesis (UDP-glucose:flavonoid 3-O-glucosyltransferase, cluster 4) were induced, while those involved in tannins synthesis (anthocyanidin reductase and leucoanthocyanidin reductase) were repressed. The profiles of genes coding for phenylalanine ammonia lyase, chalcone isomerase 1 and flavonoid 3'5'-hydroxylase showed a decrease until véraison followed by an induction during ripening, confirming previous data [75]. Two isoforms of 4-coumarate-CoA ligase with opposite profiles were detected (clusters 3 and 8) and their pattern of expression is consistent with an involvement into the anthocyanin and lignin synthesis pathways, respectively.

Eleven members of the ATP-binding cassette (ABC) and multidrug and toxic compound extrusion (MATE) transporters families which are involved in several cellular transport processes (reviewed in [76]), were modulated. Among them, the three transporters positively modulated during berry development (1610275_at, 1607763_at, 1616929_at) represent possible candidates for secondary metabolites transport.

### Gene expression profiles highly influenced by seasonal variation

**Figure 8:**
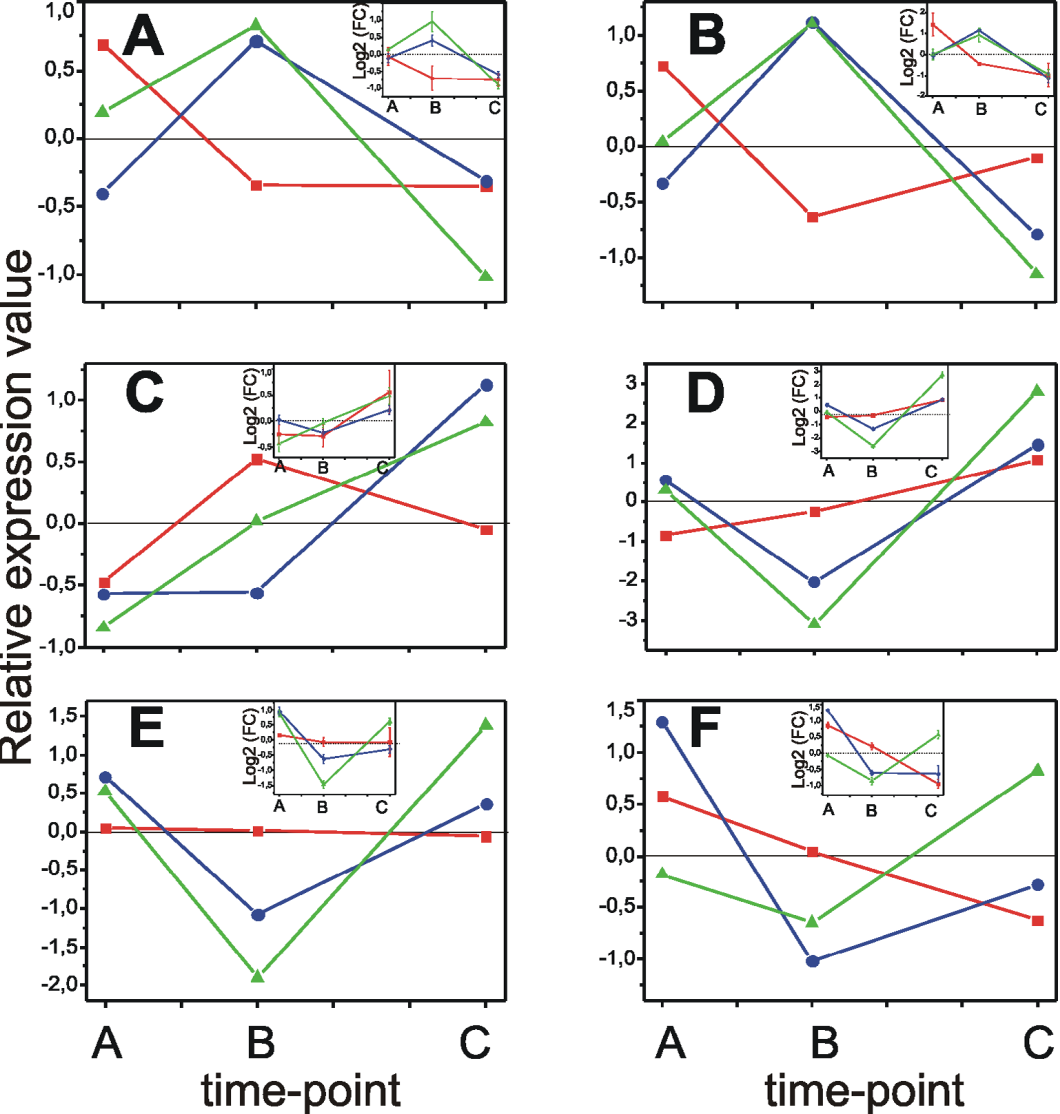
**Real time RT-PCR validation of the expression profiles of six genes highly influenced by the season**. A: 1615199_at, Cryptochrome 1; B: 1616872_at, Pseudo response regulator 9; C: 1614764_at, VvHT2, hexose transporter 2; D: 1606663_at, VvChs3, chalcone synthase isoform 3; E: 1607732_at, VvChs2: chalcone synthase isoform 2; F: 1620424_at, VvChi2, chalcone isomerase isofom 2. 2003 profiles are represented in red, 2005 in blue and 2006 in green. Relative expression data of the array hybridization experiments were centered on the average of the log_2 _values of each season. Expression profiles measured by RT-PCR experiments (insets) were first centered on the mean Ct value calculated on the three seasons for each gene and then log_2 _transformed. RT-PCR data are reported as means ± SE of three technical replicates.

The influence on gene expression by environmental conditions is well reported in literature [77] and was evident in our PCA analysis (Fig. 2). The comparison of the three sampling years highlighted a large number of genes modulated only during one or two seasons, and thus excluded by the core set of ripening-specific genes: 938 genes in 2003, 2530 in 2005 and 2143 in 2006. Two examples are the isoform 2 of the phenylpropanoid biosynthetic genes chalcone synthase and chalcone isomerase whose expression profiles are depicted in Fig. 8E and 8F; such discrepancy in their modulation is indeed expected for non-ripening specific isoforms. Although these genes are probably highly affected by changes in the environment their GO functional categories distribution was not significantly different to that of the core set genes (data not shown). This result suggests that, on a long time frame, the plant reacts to the seasonal variations by adjusting the whole metabolism, and not just a part of it, to maintain homeostasis.

The group of genes modulated in all three seasons with different transcriptional profiles, deserves in our opinion a special discussion as they are likely involved into the ripening process but in the meantime highly influenced by the climatic conditions. These 223 genes represent about 13% of the modulated gene set (1700 transcripts). Among them, at least four transcripts are putatively involved in light perception and signalling: cryptochrome 1 (1615199_at) and pseudo-response regulator 9 (APRR9, 1616872_at) showed opposite behaviours in 2003 vs. 2005 and 2006 (Fig. 8A and 8B); pseudo-response-regulator 7 (APRR7, 1608006_at) displayed a "V" profile in 2003 and 2005 and the opposite pattern during 2006; finally LONG HYPOCOTYL 5 (HY5, 1609930_at) showed a different profile from TP A to TP B in 2003 and 2005 vs. 2006 while it was repressed in all three seasons from TP B to TP C. Interestingly, APRR7 and APRR9 seem involved in the temperature responsiveness of the *Arabidopsis *circadian clock, synchronizing photo- and thermo-cycles [78] and HY5 is a positive regulator of light signalling which acts downstream of several photoreceptors and mediates the response to different types of light (red/far red, blue and UV-B [79,80]).

Out of the three previously characterized grape hexose transporters present on the chip, two (Vvht1 and Vvht2) seemed influenced by the season. Vvht1 (1616083_at) showed a "V" profile in all the three years, but with different slopes, suggesting a strong environmental conditioning along with the reported cultivar specificity [10,81,82]. Unlike Vvht1, Vvht2 is considered a ripening specific isoform [10,82,83]; however, the present work highlighted a certain degree of variability in its induction time in the three years (1614764_at, Fig. 8C). A similar behaviour was observed for another ripening specific gene, namely the isoform 3 of the chalcone synthase family [75,84] (Fig. 8D).

To ensure that the observed differences among seasons and among gene isoforms were not technical artefacts, the expression profile of six genes was validated by real time RT-PCR (Fig. 8, insets). In all but one case (Vvht2 year 2003) there was complete agreement with the array data.

## Conclusion

The time-course gene expression analysis of Pinot Noir berry development, based on a systematic and genome-wide approach, has identified the occurrence of two well distinct phases along the process. The pre-véraison phase represents a reprogramming stage of the cellular metabolism, characterized by the expression of numerous genes involved in hormonal signalling and transcriptional regulation. The post-véraison phase is characterized by the onset of a ripening-specialized metabolism responsible for the phenotypic traits of the ripe berry. A similar switch in fruit metabolism has been recently reported also in Citrus [16]. Five functional categories are overrepresented in this ripening-specialized metabolism: cell wall, carbohydrate and secondary metabolisms, stress response and photosynthesis; the latter being obviously switched off. An oxidative burst, previously not detected in grapevine and characterized by a rapid accumulation of H_2_O_2 _starting from véraison, was demonstrated. A concurrent modulation of the enzymatic ROS scavenging network was also highlighted, suggesting a possible role of ROS species in the regulation of berry ripening. We have found a very large number of transcription factors and transcripts related to hormonal metabolism and signal transduction that appear to be regulated during berry development. These represent 19% of the modulated gene set, corroborating the widely-accepted hypothesis that fruit development is under tight transcriptional control. These data provide the basis for subsequent correlation analysis aimed at highlighting gene co-regulation and metabolic networks [85]. Our strategy to perform the experiment during three seasons allowed us to restrict the dataset of modulated genes to those more likely to be ripening-specific. Nonetheless, we were able to separate ripening specific isoforms within gene families and to identify ripening related genes which appeared strongly regulated also by the seasonal weather conditions.

In perspective, the wealth of information we present here will provide a new extended platform to study the complex process of grapevine berry development and more in general of non-climacteric fruits.

## Methods

### Plant Material and Experimental Design

Ten grape clusters of *Vitis vinifera *cv. Pinot Noir were collected weekly between 8 a.m and 9 a.m, from flowering to over-ripening during 2003, 2005 and 2006 seasons at the IASMA study site 'ai Molini' (San Michele all'Adige-TN Italy). To be more representative of the canopy five clusters were collected from the north side (shady) and the other five from the south side (sunny). Berries were rapidly detached with their petioles and divided randomly into groups of 50 that were either immediately frozen for subsequent transcriptional analysis or subjected to biochemical analyses, including must acid and sugar content, average berry weight determination and anthocyanin concentration [68]. Based on these parameters, the peak of acidity just before véraison was estimated and two additional time-points (two weeks before and three weeks after véraison) were chosen for gene expression analysis. The three TPs correspond to the 33, 34 and 36 of the modified E-L system [12]. For each time-point three biological replicates were obtained by performing an independent RNA extraction from three sub-pools of 8 berries derived from the group of 50 berries. Consequently nine arrays were utilized for each season.

### RNA Preparation, Array Hybridization, Data Analysis

Total RNA was extracted from frozen deseeded berries according to [86] and further purified using RNeasy spin columns (Qiagen). RNA quality and quantity were assessed by gel-electrophoresis and by absorbance measurements. Ten μg of each sample were sent to the IFOM (Milan-I) Affymetrix platform facility for probe synthesis, GeneChip^® ^Vitis arrays hybridization, washing, staining and scanning with GeneChip Scanner 3000, according to the 'Affymetrix GeneChip^® ^Expression Analysis Technical Manual'. Data referred to 2003, 2005 and 2006 were analyzed independently in order to obtain one set of differentially expressed genes for each season. Bioconductor packages [87] in R v.2.1.0 [88] were used for the statistical analyses. Data were pre-processed using the GCRMA method [89], which performs background correction, quantile normalization and summarization, and returns log2 converted expression values. Biological replicates quality was assessed by means of R-squared coefficient, which represents the fraction of variance explained by a linear model. For variation assessment of the gene expression values between all 27 data sets, data from the three seasons were scaled and Principal Components Analysis (PCA) was performed. In order to identify genes modulated during berry development, a filtering step based on the inter-quantile range method (IQR = 0.25) was used. This restricted dataset, enriched in differentially expressed genes, was suited to run a multiclass comparison method of Significance Analysis of Microarrays (SAM), with false discovery rate =0.15% [13]. SAM output was further restricted to genes with fold-change greater than 2 in at least one of the two comparisons (TP A vs. TP B and TP B vs. TP C). The final datasets relative to the three seasons were intersected obtaining a dataset of 1700 common modulated genes. Pearson's correlation coefficients between genes profiles relative each pair of seasons were then calculated and a threshold of 0.5 was fixed to obtain a berry ripening-modulated core dataset of 1477 genes. Expression profiles clustering was carried out by the k-means method (k = 12) with Pearson's correlation distance using the T-MeV software [90]. The number of clusters was manually reduced to eight, which represents the minimum number of expression profiles considering three time-points.

### Affymetrix Vitis Array

The GeneChip^® ^*Vitis vinifera *genome array (Affymetrix) consists of 16,436 probesets: 14,496 derived from *V. vinifera *transcripts and 1,940 derived from other *Vitis *species or hybrids transcripts. It can interrogate 12,908 GenBank accessions of *V. vinifera *and 1,547 of other *Vitis *species or hybrids. Sequences used in the design of the Vitis GeneChip^® ^were selected from GenBank, dbEST, and RefSeq. The sequence clusters were created from the UniGene database (Build 7, Oct 2003). *V. vinifera *sequences represented on the chip correspond to 10,042 TIGR Tentative Consensus and 1,940 Singletons (Release 4, Sept 2004), while 102 GenBank accessions are not present in the TIGR database. Overall chip redundancy is estimated to be 16.6%.

### Annotation

The consensus sequence of each Vitis GeneChip^® ^probeset (available at [91] was analyzed to identify the coding sequence (cds) by means of an in-house developed method, which combines the results of two cds predictors: Estscan [92] and FrameFinder (at ESTate, [93]). 13,923 cds were obtainded and used to interrogate the Uniprot database (Sept. '05) by means of 'blastp', while the remaining 2,513 sequences were used to interrogate the same database by means of 'blastx' [94]. Blast results (E-value <e-10) with GO associated terms ([14]) were analyzed by the in-house developed program 'GORetriever' for annotation and 12,242 sequences were annotated with high confidence (> 80%). The automatic annotation results for the 1700 modulated genes were manually inspected and integrated with GO 'biological process' terms supported by literature evidences.

Genes were grouped into 17 functional categories based on GO 'biological process' terms by means of the in-house developed program 'GOSlimer' [see Additional file 4 for the list of the GO categories and Additional file 3 for genes annotation]. Functional categories distribution in the modulated and chip sequences were compared by means of Chi square and Fisher statistical tests (p-value < 0.001).

### Determination of the H_2_O_2 _content

H_2_O_2 _content was measured by a fluorimetric assay based on the substrate 10-acetyl-3,7-dihydroxyphenoxazine which is oxidized in the presence of H_2_O_2 _and peroxidase, using the Amplex Red Hydrogen Peroxide/Peroxidase Assay (Molecular Probes, Eugene, USA) following the manufacturer's instructions. Ten frozen berries for each time point considered were ground in liquid nitrogen and 0.2 g of fine powder were then solubilized, for each replicate, with 0.5 ml of 50 mM phosphate buffer (pH 7.4) and kept 5 min on ice. After centrifugation at 20,000 g for 15 min, the cleared supernatant was extracted with an equal volume of 2:1 (v/v) chloroform:methanol mixture and centrifuged at 12,000 g for 5 min. To perform the H_2_O_2 _assay 50 μl of the aqueous phase were added to 50 μl of the reagent working solution. After 30 min of incubation at 25°C, relative fluorescence (excitation at 544 nm and emission at 590 nm) was measured in a black 96 wells plate. Absolute quantification was determined by the use of a H_2_O_2 _standard curve.

### Real time RT-PCR

First strand cDNA synthesis was performed on one mRNA sample of the three biological replicates used for the microarray experiments using the SuperScript™ III Reverse Transcriptase kit (Invitrogen) according to the manufacturer instructions. Primers [see Additional file 8 for sequences] and cDNA were mixed with the Platinum^® ^SYBR^® ^Green qPCR SuperMix-UDG (Invitrogen) and the reaction was carried out on an ABI PRISM 7000 Sequence Detection System (Applied Biosystems). Cycling conditions were: 50°C for 2 minutes, 95°C for 2 minutes, then 40 cycles of 95°C for 15 seconds and 60°C for 1 minute. Raw data were analyzed with ABI PRISM 7000 SDS software to extract Ct values and with the LinReg software to calculate the reaction efficiency [95]. Relative expression of each gene (target) was then calculated according to the equation by [96] using actin for normalization (reference) and centered on the mean Ct calculated on the three seasons and on the three time points (control): RelExp = E_(target)_exp[ΔCt_(target)_(sample-control)]/E_(reference)_exp[ΔCt_(reference)_(sample-control)].

### Data Availability

All microarray expression data produced by this work are available at EBI ArrayExpress [97] under the series entry E-MEXP-1282.

## Abbreviations

cDNA Complementary DNA

EST Expressed Sequence Tag

GO Gene Ontology

NCBI National Center for Biotechnology Information

SAM Significance Analysis of Microarrays

TIGR The Institute for Genomic Research

TAIR The *Arabidopsis *Information Resource

## Authors' contributions

SP has made substantial contribution to conception, data analysis and manuscript drafting. MP participated in the GO annotation of the sequences, performed profiles clustering and revised critically the manuscript. AM has contributed to data analysis and revised critically the manuscript. AC, LD and PF contributed to sequence annotation. ADR carried out samples collection, RNA isolation, the biochemical assays and the real time PCR experiments. RV contributed to critically revise the manuscript. RV participated to project's design and coordination. CM has made substantial contribution to conception, project coordination and helped in drafting the manuscript. All authors read and approved the final manuscript.

## Supplementary Material

Additional file 1 Winkler index. Winkler index of the study site calculated for the seasons 2002–2006. Click here for file

Additional file 2 Data quality assessment. R-squared coefficient analysis on the 27 hybridization data sets. Click here for file

Additional file 3 Core set of modulated genes during berry ripening. Log_2 _average expression data for 2003, 2005 and 2006 of the 1477 modulated genes during berry ripening. Some more information such as the corresponding TC or singleton of the TIGR gene index v 4.0,, the expression profile cluster, the sequence annotation and the corresponding GO functional category are also included. Click here for file

Additional file 4 List of the GO functional categories. Gene Ontology codes with obsolete and updated descriptions of the functional categories used in this work. Click here for file

Additional file 5 Functional categories distribution in the Vitis GeneChip^®^. Comparison of the functional categories distribution in the Affymetrix Vitis GeneChip and in the *Arabidopsis *genome. Click here for file

Additional file 6 Functional categories distribution in the expression clusters. Functional categories distribution in the eight clusters obtained by the k-means method on the gene expression profiles of the 1477 modulated genes. Click here for file

Additional file 7 Complete list of genes belonging to the functional categories discussed in the article. Genes of the core set involved in the regulation of berry development (part A) and responsible for berry phenotypic traits (part B), ordered by major GO functional categories (in red). For clarity, transcripts discussed in the manuscript were further classified according to GO more specific terms. Click here for file

Additional file 8 List of the primers used for the RT-PCR validation experiment. Sequence of the primers used in real time reverse transcription-polymerase chain reaction. Click here for file

## References

[B1] Doligez A, Adam-Blondon AF, Cipriani G, Di Gaspero G, Laucou V, Merdinoglu D, Meredith CP, Riaz S, Roux C, This P (2006). An integrated SSR map of grapevine based on five mapping populations. Theor Appl Genet.

[B2] IASMA Genomics. http://genomics.research.iasma.it.

[B3] NCBI dbest. http://www.ncbi.nlm.nih.gov/dbEST/.

[B4] Coombe BG, Mccarthy MG (2000). Dynamics of Grape Berry Growth and Physiology of Ripening. Aust J Grape Wine Res.

[B5] Ablett E, Seaton G, Scott K, Shelton D, Graham MW, Baverstock P, Lee LS, Henry R (2000). Analysis of grape ESTs: global gene expression patterns in leaf and berry. Plant Sci.

[B6] Moser C, Segala C, Fontana P, Salakhudtinov I, Gatto P, Pindo M, Zyprian E, Toepfer R, Grando MS, Velasco R (2005). Comparative analysis of expressed sequence tags from different organs of *Vitis vinifera *L. Funct Integr Genomics.

[B7] da Silva FG, Iandolino A, Al-Kayal F, Bohlmann MC, Cushman MA, Lim H, Ergul A, Figueroa R, Kabuloglu EK, Osborne C, Rowe J, Tattersall E, Leslie A, Xu J, Baek J, Cramer GR, Cushman JC, Cook DR (2005). Characterizing the grape transcriptome. Analysis of expressed sequence tags from multiple *Vitis *species and development of a compendium of gene expression during berry development. Plant Physiol.

[B8] Davies C, Robinson SP (2000). Differential screening indicates a dramatic change in mRNA profiles during grape berry ripening. Cloning and characterization of cDNAs encoding putative cell wall and stress response proteins. Plant Physiol.

[B9] Waters DL, Holton TA, Ablett EM, Lee LS, Henry RJ (2005). cDNA microarray analysis of developing grape (*Vitis vinifera *cv. Shiraz) berry skin. Funct Integr Genomics.

[B10] Terrier N, Glissant D, Grimplet J, Barrieu F, Abbal P, Couture C, Ageorges A, Atanassova R, Leon C, Renaudin JP, Dedaldechamp F, Romieu C, Delrot S, Hamdi S (2005). Isogene specific oligo arrays reveal multifaceted changes in gene expression during grape berry (*Vitis vinifera *L.) development. Planta.

[B11] Grimplet J, Deluc LG, Tillett RL, Wheatley MD, Schlauch KA, Cramer GR, Cushman JC (2007). Tissue-specific mRNA expression profiling in grape berry tissues. BMC Genomics.

[B12] Coombe BG (1995). Adoption of a system for identifying grapevine growth stages. Aust J Grape Wine Res.

[B13] Tusher VG, Tibshirani R, Chu G (2001). Significance analysis of microarrays applied to the ionizing radiation response. Proc Natl Acad Sci USA.

[B14] Gene Ontology home. http://www.geneontology.org/.

[B15] TAIR:The *Arabidopsis *Information Resource. http://www.arabidopsis.org.

[B16] Cercos M, Soler G, Iglesias DJ, Gadea J, Forment J, Talon M (2006). Global analysis of gene expression during development and ripening of citrus fruit flesh. A proposed mechanism for citric acid utilization. Plant Mol Biol.

[B17] Alba R, Payton P, Fei Z, McQuinn R, Debbie P, Martin GB, Tanksley SD, Giovannoni JJ (2005). Transcriptome and selected metabolite analyses reveal multiple points of ethylene control during tomato fruit development. Plant Cell.

[B18] Coombe BG, Hale CR (1973). The hormone content of ripening grape berries and the effects of growth substance treatments. Plant Physiol.

[B19] Davies C, Boss PK, Robinson SP (1997). Treatment of grape berries, a nonclimacteric fruit with a synthetic auxin, retards ripening and alters the expression of developmentally regulated genes. Plant Physiol.

[B20] Aharoni A, Keizer LC, Van Den Broeck HC, Blanco-Portales R, Munoz-Blanco J, Bois G, Smit P, De Vos RC, O'Connell AP (2002). Novel insight into vascular, stress, and auxin-dependent and -independent gene expression programs in strawberry, a non-climacteric fruit. Plant Physiol.

[B21] Gillaspy G, Ben-David H, Gruissem W (1993). Fruits: a developmental perspective. Plant Cell.

[B22] Cawthon DL, Morris JR (1982). Relationship of seed number and maturity to berry development, fruit maturation, hormonal changes, and uneven berry ripening in 'Concord' (*Vitis labrusca*) grapes. J Am Soc Hortic Sci.

[B23] Symons GM, Davies C, Shavrukov Y, Dry IB, Reid JB, Thomas MR (2006). Grapes on steroids. Brassinosteroids are involved in grape berry ripening. Plant Physiol.

[B24] Pollmann S, Neu D, Weiler EW (2003). Molecular cloning and characterization of an amidase from *Arabidopsis thaliana *capable of converting indole-3-acetamide into the plant growth hormone, indole-3-acetic acid. Phytochemistry.

[B25] Staswick PE, Serban B, Rowe M, Tiryaki I, Maldonado MT, Maldonado MC, Suza W (2005). Characterization of an *Arabidopsis *enzyme family that conjugates amino acids to indole-3-acetic acid. Plant Cell.

[B26] Liu K, Kang BC, Jiang H, Moore SL, Li H, Watkins CB, Setter TL, Jahn MM (2005). A GH3-like gene, CcGH3, isolated from *Capsicum chinense *L. fruit is regulated by auxin and ethylene. Plant Mol Biol.

[B27] Hale CR, Coombe BG, Hawker JS (1970). Effects of ethylene and 2-chloroethylphosphonic acid on the ripening of grapes. Plant Physiol.

[B28] Trainotti L, Pavanello A, Casadoro G (2005). Different ethylene receptors show an increased expression during the ripening of strawberries: does such an increment imply a role for ethylene in the ripening of these non-climacteric fruits?. J Exp Bot.

[B29] Chervin C, El-Kereamy A, Roustan JP, Latche A, Lamon J, Bouzayen M (2004). Ethylene seems required for the berry development and ripening in grape, a non-climacteric fruit. Plant Sci.

[B30] Tournier B, Sanchez-Ballesta MT, Jones B, Pesquet E, Regad F, Latche A, Pech JC, Bouzayen M (2003). New members of the tomato ERF family show specific expression pattern and diverse DNA-binding capacity to the GCC box element. FEBS Lett.

[B31] Lorenzo O, Piqueras R, Sanchez-Serrano JJ, Solano R (2003). ETHYLENE RESPONSE FACTOR1 integrates signals from ethylene and jasmonate pathways in plant defense. Plant Cell.

[B32] Ohme-Takagi M, Shinshi H (1995). Ethylene-inducible DNA binding proteins that interact with an ethylene-responsive element. Plant Cell.

[B33] Xu P, Narasimhan ML, Samson T, Coca MA, Huh GH, Zhou J, Martin GB, Hasegawa PM, Bressan RA (1998). A nitrilase-like protein interacts with GCC box DNA-binding proteins involved in ethylene and defense responses. Plant Physiol.

[B34] Zegzouti H, Jones B, Frasse P, Marty C, Maitre B, Latch A, Pech JC, Bouzayen M (1999). Ethylene-regulated gene expression in tomato fruit: characterization of novel ethylene-responsive and ripening-related genes isolated by differential display. Plant J.

[B35] Kneissl ML, Deikman J (1996). The tomato E8 gene influences ethylene biosynthesis in fruit but not in flowers. Plant Physiol.

[B36] Aharoni A, O'Connell AP (2002). Gene expression analysis of strawberry achene and receptacle maturation using DNA microarrays. J Exp Bot.

[B37] Giovannoni JJ (2004). Genetic regulation of fruit development and ripening. Plant Cell.

[B38] Zeevaart JAD, Creelman RA (1988). Metabolism and physiology of abscisic-acid. Annu Rev Plant Physiol Plant Mol Biol.

[B39] Xiao H, Nassuth A (2006). Stress- and development-induced expression of spliced and unspliced transcripts from two highly similar dehydrin 1 genes in *V. riparia *and *V. vinifera*. Plant Cell Rep.

[B40] Jang JY, Kim DG, Kim YO, Kim JS, Kang H (2004). An expression analysis of a gene family encoding plasma membrane aquaporins in response to abiotic stresses in *Arabidopsis thaliana*. Plant Mol Biol.

[B41] Montoya T, Nomura T, Yokota T, Farrar K, Harrison K, Jones JD, Kaneta T, Kamiya Y, Szekeres M, Bishop GJ (2005). Patterns of Dwarf expression and brassinosteroid accumulation in tomato reveal the importance of brassinosteroid synthesis during fruit development. Plant J.

[B42] Nakamichi N, Murakami-Kojima M, Sato E, Kishi Y, Yamashino T, Mizuno T (2002). Compilation and characterization of a novel WNK family of protein kinases in Arabiodpsis thaliana with reference to circadian rhythms. Biosci Biotechnol Biochem.

[B43] Kikis EA, Khanna R, Quail PH (2005). ELF4 is a phytochrome-regulated component of a negative-feedback loop involving the central oscillator components CCA1 and LHY. Plant J.

[B44] Kardailsky I, Shukla VK, Ahn JH, Dagenais N, Christensen SK, Nguyen JT, Chory J, Harrison MJ, Weigel D (1999). Activation tagging of the floral inducer FT. Science.

[B45] Hwang I, Chen HC, Sheen J (2002). Two-component signal transduction pathways in *Arabidopsis*. Plant Physiol.

[B46] Ageorges A, Fernandez L, Vialet S, Merdinoglu D, Terrier N, Romieu C (2006). Four specific isogenes of the anthocyanin metabolic pathway are systematically co-expressed with the red colour of grape berries. Plant Sci.

[B47] Kobayashi S, Ishimaru M, Hiraoka K, Honda C (2002). Myb-related genes of the Kyoho grape (*Vitis labruscana*) regulate anthocyanin biosynthesis. Planta.

[B48] Deluc L, Barrieu F, Marchive C, Lauvergeat V, Decendit A, Richard T, Carde JP, Merillon JM, Hamdi S (2006). Characterization of a grapevine R2R3-MYB transcription factor that regulates the phenylpropanoid pathway. Plant Physiol.

[B49] Boss PK, Buckeridge EJ, Poole A, Thomas MR (2003). New insights into grapevine flowering. Funct Plant Biol.

[B50] Boss PK, Vivier M, Matsumoto S, Dry IB, Thomas MR (2001). A cDNA from grapevine (*Vitis vinifera *L.), which shows homology to AGAMOUS and SHATTERPROOF, is not only expressed in flowers but also throughout berry development. Plant Mol Biol.

[B51] Vrebalov J, Ruezinsky D, Padmanabhan V, White R, Medrano D, Drake R, Schuch W, Giovannoni J (2002). A MADS-box gene necessary for fruit ripening at the tomato ripening-inhibitor (rin) locus. Science.

[B52] Olsen AN, Ernst HA, Leggio LL, Skriver K (2005). NAC transcription factors: structurally distinct, functionally diverse. Trends Plant Sci.

[B53] Ulker B, Somssich IE (2004). WRKY transcription factors: from DNA binding towards biological function. Curr Opin Plant Biol.

[B54] Paquette AJ, Benfey PN (2005). Maturation of the ground tissue of the root is regulated by gibberellin and SCARECROW and requires SHORT-ROOT. Plant Physiol.

[B55] Cakir B, Agasse A, Gaillard C, Saumonneau A, Delrot S, Atanassova R (2003). A grape ASR protein involved in sugar and abscisic acid signaling. Plant Cell.

[B56] Atanassova R, Leterrier M, Gaillard C, Agasse A, Sagot E, Coutos-Thevenot P, Delrot S (2003). Sugar-regulated expression of a putative hexose transport gene in grape. Plant Physiol.

[B57] Sarry JE, Sommerer N, Sauvage FX, Bergoin A, Rossignol M, Albagnac G, Romieu C (2004). Grape berry biochemistry revisited upon proteomic analysis of the mesocarp. Proteomics.

[B58] Robinson SP, Davies C (2000). Molecular biology of grape berry ripening. Aust J Grape Wine Res.

[B59] Nunan KJ, Davies C, Robinson SP, Fincher GB (2001). Expression patterns of cell wall-modifying enzymes during grape berry development. Planta.

[B60] Saladie M, Rose JK, Cosgrove DJ, Catala C (2006). Characterization of a new xyloglucan endotransglucosylase/hydrolase (XTH) from ripening tomato fruit and implications for the diverse modes of enzymic action. Plant J.

[B61] Jimenez A, Creissen G, Kular B, Firmin J, Robinson S, Verhoeyen M, Mullineaux P (2002). Changes in oxidative processes and components of the antioxidant system during tomato fruit ripening. Planta.

[B62] Meir S, Philosoph-Hadas S, Zauberman G, Fuchs Y, Akerman M, Aharoni N (1991). Increased formation of fluorescent lipid-peroxidation products in avocado peels precedes other signs of ripening. J Amer Soc Hort Sci.

[B63] Moyle R, Fairbairn DJ, Ripi J, Crowe M, Botella JR (2005). Developing pineapple fruit has a small transcriptome dominated by metallothionein. J Exp Bot.

[B64] Brennan T, Frenkel C (1977). Involvement of hydrogen peroxide in the regulation of senescence in pear. Plant Physiol.

[B65] Mittler R, Vanderauwera S, Gollery M, Van Breusegem F (2004). Reactive oxygen gene network of plants. Trends Plant Sci.

[B66] Ogawa T, Ueda Y, Yoshimura K, Shigeoka S (2005). Comprehensive analysis of cytosolic Nudix hydrolases in *Arabidopsis thaliana*. J Biol Chem.

[B67] Terrier N, Romieu C (2001). Grape berry acidity.

[B68] Cheng GW, Breen PJ (1991). Activity of phenylalanine ammonia-lyase (PAL) and concentrations of anthocyanins and phenolics in developing strawberry fruit. J Am Soc Hortic Sci.

[B69] Zhang X-Y, Wang X-L, Wang X-F, Xia G-H, Pan Q-H, Fan R-C, Wu F-Q, Yu X-C, Zhang D-P (2006). A shift of phloem unloading from symplasmic to apoplasmic pathway is involved in developmental onset of ripening in grape berry. Plant Physiol.

[B70] Davies C, Wolf T, Robinson SP (1999). Three putative sucrose transporters are differentially expressed in grapvine tissues. Plant Sci.

[B71] Davies C, Robinson SP (1996). Sugar accumulation in grape berries. Cloning of two putative vacuolar invertase cDNAs and their expression in grapevine tissues. Plant Physiol.

[B72] Nguyen-Quoc B, Foyer CH (2001). A role for 'futile cycles' involving invertase and sucrose synthase in sucrose metabolism of tomato fruit. J Exp Bot.

[B73] Terrier N, Sauvage FX, Ageorges A, Romieu C (2001). Changes in acidity and in proton transport at the tonoplast of grape berries during development. Planta.

[B74] Mellema S, Eichenberger W, Rawyler A, Suter M, Tadege M, Kuhlemeier C (2002). The ethanolic fermentation pathway supports respiration and lipid biosynthesis in tobacco pollen. Plant J.

[B75] Boss PK, Davies C, Robinson SP (1996). Analysis of the expression of anthocyanin pathway genes in developing *Vitis vinifera *L. cv Shiraz grape berries and the implications for pathway regulation. Plant Physiol.

[B76] Martinoia E, Klein M, Geisler M, Bovet L, Forestier C, Kolukisaoglu U, Muller-Rober B, Schulz B (2002). Multifunctionality of plant ABC transporters – more than just detoxifiers. Planta.

[B77] Wissel K, Pettersson F, Berglund A, Jansson S (2003). What affects mRNA levels in leaves of field-grown aspen? A study of developmental and environmental influences. Plant Physiol.

[B78] Salome PA, McClung CR (2005). PSEUDO-RESPONSE REGULATOR 7 and 9 are partially redundant genes essential for the temperature responsiveness of the *Arabidopsis *circadian clock. Plant Cell.

[B79] Ulm R, Baumann A, Oravecz A, Mate Z, Adam E, Oakeley EJ, Schafer E, Nagy F (2004). Genome-wide analysis of gene expression reveals function of the bZIP transcription factor HY5 in the UV-B response of *Arabidopsis*. Proc Natl Acad Sci USA.

[B80] Tepperman JM, Zhu T, Chang HS, Wang X, Quail PH (2001). Multiple transcription-factor genes are early targets of phytochrome A signaling. Proc Natl Acad Sci USA.

[B81] Conde C, Agasse A, Glissant D, Tavares R, Geros H, Delrot S (2006). Pathways of glucose regulation of monosaccharide transport in grape cells. Plant Physiol.

[B82] Fillion L, Ageorges A, Picaud S, Coutos-Thevenot P, Lemoine R, Romieu C, Delrot S (1999). Cloning and expression of a hexose transporter gene expressed during the ripening of grape berry. Plant Physiol.

[B83] Vignault C, Vachaud M, Cakir B, Glissant D, Dedaldechamp F, Buttner M, Atanassova R, Fleurat-Lessard P, Lemoine R, Delrot S (2005). VvHT1 encodes a monosaccharide transporter expressed in the conducting complex of the grape berry phloem. J Exp Bot.

[B84] Goto-Yamamoto N, Wan GH, Masaki K, Kobayashi S (2002). Structure and transcription of three chalcone synthase genes of grapevine (*Vitis vinifera*). Plant Sci.

[B85] Harmer SL, Hogenesch JB, Straume M, Chang HS, Han B, Zhu T, Wang X, Kreps JA, Kay SA (2000). Orchestrated transcription of key pathways in *Arabidopsis *by the circadian clock. Science.

[B86] Moser C, Gatto P, Moser M, Pindo M, Velasco R (2004). Isolation of functional RNA from small amounts of different grape and apple tissues. Mol Biotechnol.

[B87] Gentleman RC, Carey VJ, Bates DM, Bolstad B, Dettling M, Dudoit S, Ellis B, Gautier L, Ge Y, Gentry J, Hornik K, Hothorn T, Huber W, Iacus S, Irizarry R, Leisch F, Li C, Maechler M, Rossini AJ, Sawitzki G, Smith C, Smyth G, Tierney L, Yang JY, Zhang J (2004). Bioconductor: open software development for computational biology and bioinformatics. Genome Biol.

[B88] Ihaka R, Gentleman R (1996). R: a language for data analysis and graphics. J Comput Graph Stat.

[B89] Wu Z, Irizarry RA (2004). Preprocessing of oligonucleotide array data. Nat Biotechno.

[B90] TM4: Microarray software suite. http://www.tm4.org/.

[B91] Affymetrix home site. http://www.affymetrix.com/index.affx.

[B92] Nadershahi A, Fahrenkrug SC, Ellis LB (2004). Comparison of computational methods for identifying translation initiation sites in EST data. BMC Bioinformatics.

[B93] ESTate. http://www.bio.net/bionet/mm/bio-soft/1999-December/021776.html.

[B94] Altschul SF, Madden TL, Schaffer AA, Zhang J, Zhang Z, Miller W, Lipman DJ (1997). Gapped BLAST and PSI-BLAST: a new generation of protein database search programs. Nucleic Acids Res.

[B95] Ramakers C, Ruijter JM, Deprez RH, Moorman AF (2003). Assumption-free analysis of quantitative real-time polymerase chain reaction (PCR) data. Neurosci Lett.

[B96] Pfaffl MW (2001). A new mathematical model for relative quantification in real-time RT-PCR. Nucleic Acids Res.

[B97] ArrayExpress home. http://www.ebi.ac.uk/microarray-as/aer/?#ae-main[0].

