# Supplementary figures and images for: Genome-wide transcriptional analysis of grapevine berry ripening reveals a set of genes similarly modulated during three seasons and the occurrence of an oxidative burst at vèraison
Source: BMC Genomics. 2007 Nov 22;8:428. doi: 10.1186/1471-2164-8-428 (PMC2228314; doi:10.1186/1471-2164-8-428)

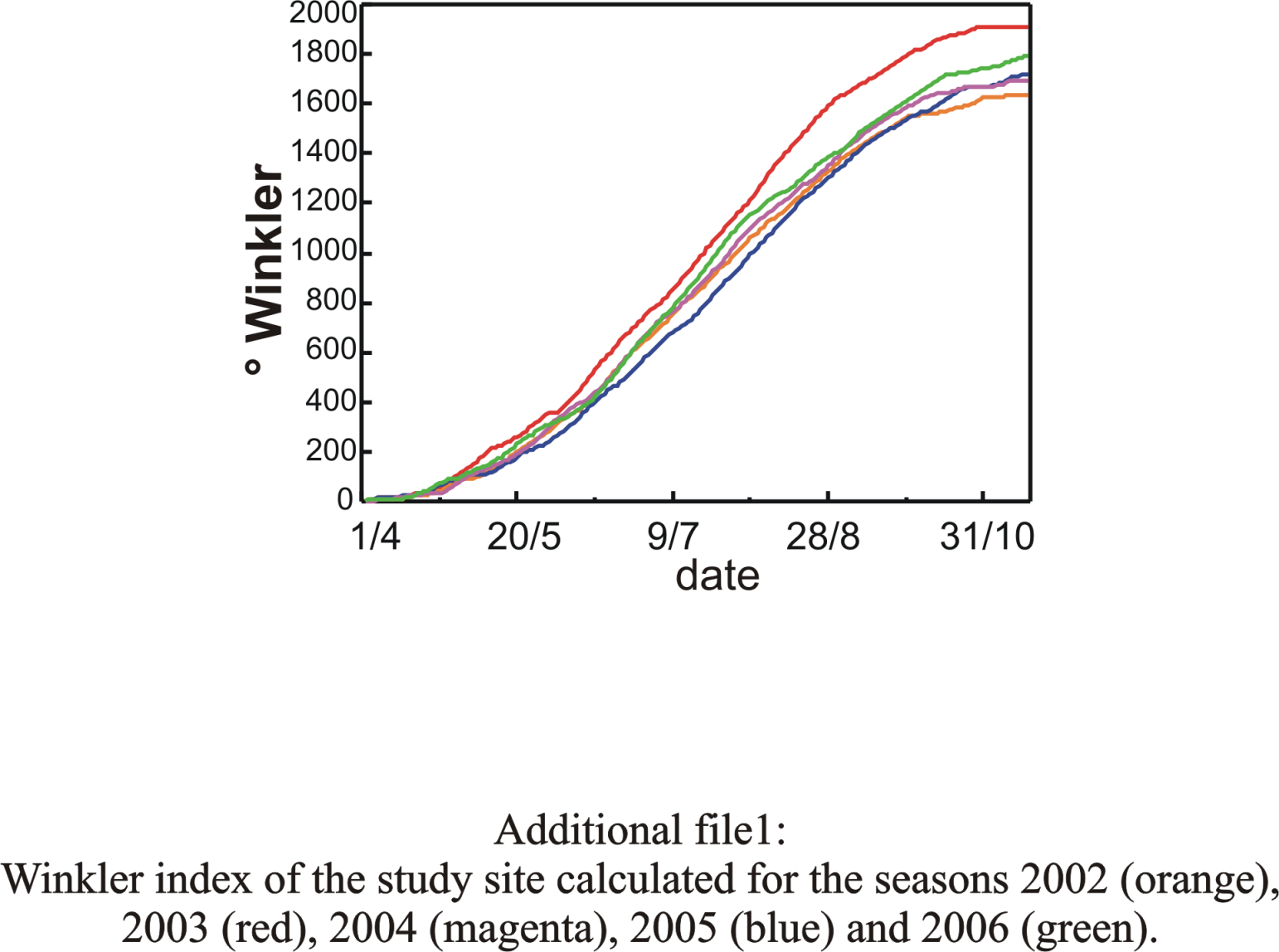

Supplement: Additional file 1 — Winkler index. Winkler index of the study site calculated for the seasons 2002–2006. [file 1471-2164-8-428-S1.tiff]

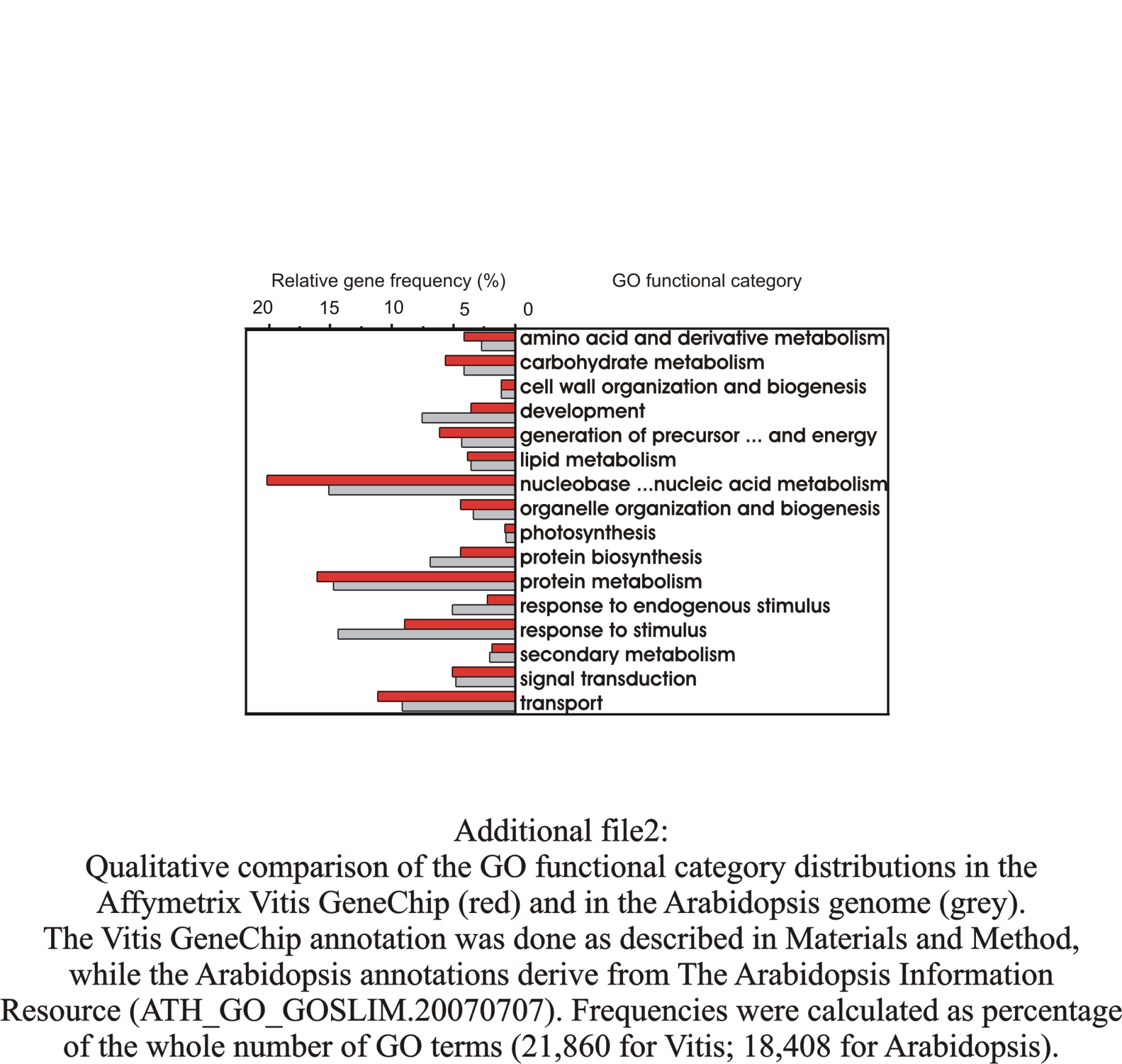

Supplement: Additional file 5 — Functional categories distribution in the Vitis GeneChip®. Comparison of the functional categories distribution in the Affymetrix Vitis GeneChip and in the Arabidopsis genome. [file 1471-2164-8-428-S5.tiff]

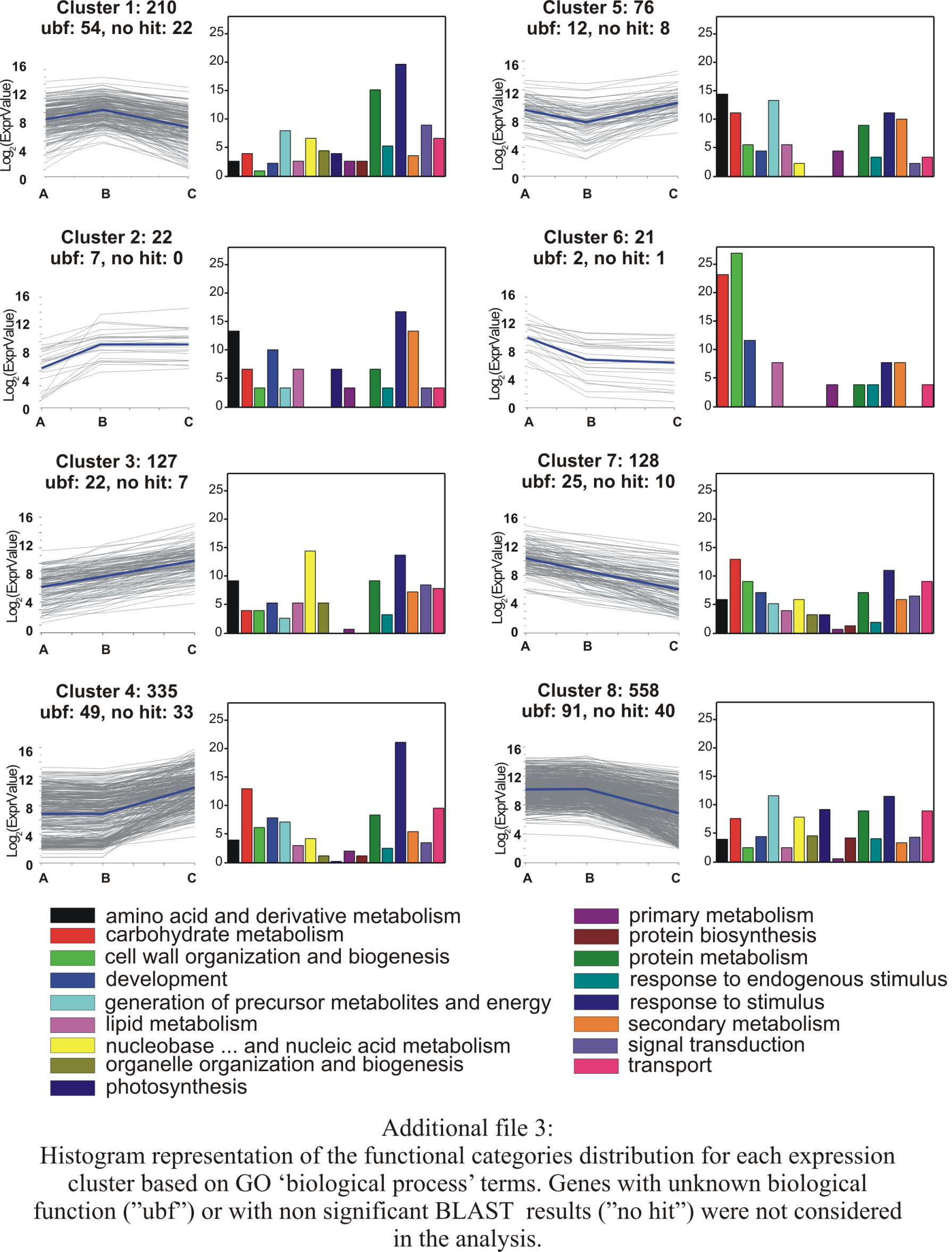

Supplement: Additional file 6 — Functional categories distribution in the expression clusters. Functional categories distribution in the eight clusters obtained by the k-means method on the gene expression profiles of the 1477 modulated genes. [file 1471-2164-8-428-S6.tiff]
