# Supplementary material for: Genome-wide transcriptional analysis of grapevine berry ripening reveals a set of genes similarly modulated during three seasons and the occurrence of an oxidative burst at vèraison
Source: BMC Genomics. 2007 Nov 22;8:428. doi: 10.1186/1471-2164-8-428 (PMC2228314; doi:10.1186/1471-2164-8-428)
Supplement: Additional file 8 — List of the primers used for the RT-PCR validation experiment. Sequence of the primers used in real time reverse transcription-polymerase chain reaction. [file 1471-2164-8-428-S8.doc]

| **Affy ID** | **Description** | **Forward primer** | **Reverse primer** |
| --- | --- | --- | --- |
| 1615199_at | Cryptochrome 1 | 5’-CCTTTTTCCACGTTTCCTGT-3’ | 5’-AGCACAAGCCTGGGTTTATT-3’ |
| 1616872_at | Pseudo response regulator 9 | 5’-TCTGGGTGTTTGGCATTG-3’ | 5’-TCGAGGCTGCACAAAAGT-3’ |
| 1614764_at | VvHT2, hexose transporter | 5’-GCCGGAGACCAAAGGAAT-3’ | 5’-GGGACTTGAGAAGGCATTTAG-3’ |
| 1606663_at | VvChs3, chalcone synthase isoform 3 | 5’-TGTTTCCGCTTTACTTGCCT-3’ | 5’-CCATACCCTTGATGCCTACC-3’ |
| 1607732_at | VvChs2, chalcone synthase isoform 2 | 5’-GAAGATGGGAATGGCTGCTG-3’ | 5’-AAGGCACAGGGACACAAAAG-3’ |
| 1620424_at | VvChi2, chalcone isomerase isoform 2 | 5’-TCATGCACTGCTGAGATTGT-3’ | 5’-GACACTCCCCTTGTTCCAC-3’ |
|  | Actin (TC45156) | 5’-TCCTTGCCTTGCGTCATCTAT-3’ | 5’-CACCAATCACTCTCCTGCTACAA-3’ |
